# CO-driven electron and carbon flux fuels synergistic microbial reductive dechlorination

**DOI:** 10.1186/s40168-024-01869-y

**Published:** 2024-08-20

**Authors:** Jingjing Wang, Xiuying Li, Huijuan Jin, Shujing Yang, Lian Yu, Hongyan Wang, Siqi Huang, Hengyi Liao, Xuhao Wang, Jun Yan, Yi Yang

**Affiliations:** 1 Key Laboratory of Pollution Ecology and Environmental Engineering, Institute of Applied Ecology, Chinese Academy of Sciences, Shenyang, Liaoning 110016 China; 2 University of Chinese Academy of Sciences, Beijing, 100049 China; 3 Shenyang Pharmaceutical University, Shenyang, Liaoning 117004 China; 4 Department of Environmental Engineering, Beijing Institute of Petrochemical Technology, Beijing, 102617 China; 5 Key Laboratory of Forest Ecology and Silviculture, Institute of Applied Ecology, Chinese Academy of Sciences, Shenyang, Liaoning 110016 China

**Keywords:** Carbon monoxide, Syntrophy, Reductive dechlorination, *Dehalococcoides*, *Acetobacterium*

## Abstract

**Background:**

Carbon monoxide (CO), hypothetically linked to prebiotic biosynthesis and possibly the origin of the life, emerges as a substantive growth substrate for numerous microorganisms. In anoxic environments, the coupling of CO oxidation with hydrogen (H_2_) production is an essential source of electrons, which can subsequently be utilized by hydrogenotrophic bacteria (e.g., organohalide-respring bacteria). While *Dehalococcoides* strains assume pivotal roles in the natural turnover of halogenated organics and the bioremediation of chlorinated ethenes, relying on external H_2_ as their electron donor and acetate as their carbon source, the synergistic dynamics within the anaerobic microbiome have received comparatively less scrutiny. This study delves into the intriguing prospect of CO serving as both the exclusive carbon source and electron donor, thereby supporting the reductive dechlorination of trichloroethene (TCE).

**Results:**

The metabolic pathway involved anaerobic CO oxidation, specifically the Wood-Ljungdahl pathway, which produced H_2_ and acetate as primary metabolic products. In an intricate microbial interplay, these H_2_ and acetate were subsequently utilized by *Dehalococcoides*, facilitating the dechlorination of TCE. Notably, *Acetobacterium* emerged as one of the pivotal collaborators for *Dehalococcoides*, furnishing not only a crucial carbon source essential for its growth and proliferation but also providing a defense against CO inhibition.

**Conclusions:**

This research expands our understanding of CO’s versatility as a microbial energy and carbon source and unveils the intricate syntrophic dynamics underlying reductive dechlorination.

**Graphical Abstract:**

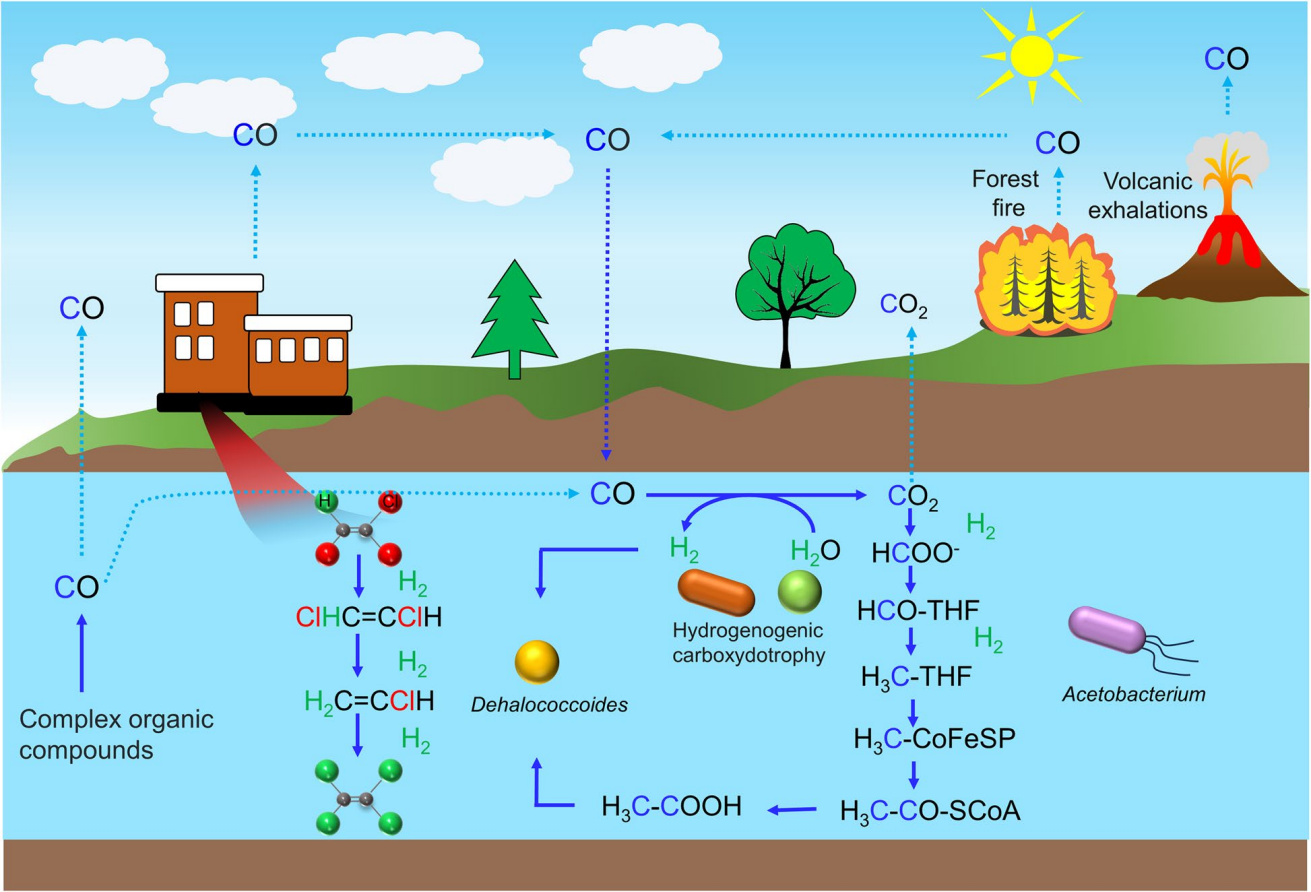

Video Abstract

**Supplementary Information:**

The online version contains supplementary material available at 10.1186/s40168-024-01869-y.

## Introduction

Carbon monoxide (CO), presumed to be abundant in the prebiotic Earth, holds compelling evidence for its involvement in prebiotic synthesis and probably the origin of life. Early life on Earth thrived amidst high levels of CO exposure [1–3], a phenomenon traced back to the primordial atmospheric conditions that prevailed around 4 billion years ago when life first emerged. The Wood-Ljungdahl pathway (WLP), an ancient carbon fixation pathway consisting of the methyl and the carbonyl branches, is hypothesized to have played a pivotal role in the origin of life on ancient Earth as well as microbial energy conservation and carbon assimilation under anoxic conditions [4–6]. CO, serving as both a carbon and electron source, finds relevance for microorganisms utilizing WLP [7, 8].

CO, once thought of as a toxic gas, has emerged as a fascinating and surprisingly versatile metabolic substrate for diverse microorganisms through both fermentative and respiratory pathways [8]. Certain aerobic bacteria, such as *Oligotropha carboxidivorans*, *Ruegeria pomeroyi*, and *Mycobacterium smegmatis* [9], are capable of oxidizing atmospheric CO, where CO serves as both electron donor and carbon source, with O_2_ as the electron acceptor. Under anoxic conditions, CO is typically metabolized through fermentative pathways, with nickel-dependent CODHs (Ni-CODH) playing a crucial role in oxidizing CO to various products, including CO_2_ plus H_2_, acetate, ethanol, or methane plus CO_2_ [7, 10, 11]. Certain groups of microorganisms, such as sulfate-reducing bacteria and iron-reducing bacteria, employ respiratory metabolism to utilize CO. In this respiratory process, CO is oxidized to CO_2_ with either SO_4_^2−^ or Fe^3+^ serving as the electron acceptor and subsequently being reduced to S^2−^ or Fe^2+^ [7, 12]. CO’s diverse metabolic pathways highlight the remarkable adaptability of microorganisms to different environmental conditions.

Organohalide-respiring bacteria (OHRB) not only gained recognition as crucial microorganisms for the bioremediation of chlorinated solvents, but it has also played pivotal roles in the natural turnover of halogenated organics [13]. *Dehalococcoides mccartyi* populations are obligate OHRB, and its noteworthy ability to convert the ubiquitous groundwater contaminants chlorinated ethenes (strains 195, FL2, BAV1, GT), chlorinated ethanes (strains 195, BAV1, VS, 11a), pentachlorophenol (strain JNA), chlorinated biphenyls (strain CG1), and chlorinated benzenes (strain CBDB1) to the low or non-toxic end products has made it a key player in environmental detoxification [14–24]. While *Dehalococcoides* excel at detoxification, their own metabolic processes, specifically the incomplete WLP for methionine biosynthesis, can turn against them, leading to CO accumulation and potentially hindering their effectiveness [25–27]. The toxicity of CO, detrimental to *Dehalococcoides* strains, could be alleviated by other CO-utilizing bacteria within the microbial community (e.g., *Desulfovibrio*, *Acetobacterium*) [10, 27–29]. CO produced by *Dehalococcoides* may also serve as an energy source for anaerobically CO-oxidizing bacteria. In addition to protections offered by other microbes (e.g., fermenters, acetogens), *Dehalococcoides* thrives optimally in microbial communities by acquiring essential resources including the electron donor H_2_ and carbon source acetate. For instance, the H_2_ and acetate, generated through lactate fermentation by *Desulfovibrio desulfuricans* strain F1, were harnessed by *Dehalococcoides* strain CG1 for organohalide respiration, highlighting a synergistic interaction [30]. CO could have served as a more thermodynamically favorable “first” electron donor than H_2_ [31] and a potential carbon source; nonetheless, evidence regarding on CO’s role in supporting reductive dechlorination remains rare. In contrast to OHRB, *Acetobacterium dehalogenans* utilizes chloromethane and produces acetate as a fermentation end product [32–34]. This distinction highlights the diverse metabolic strategies employed by microorganisms for dehalogenation. While *Acetobacterium* species are primarily known as acetogens that indirectly support dechlorination by providing essential cofactors like vitamin B_12_ and acetate [35–38], some strains, such as *Acetobacterium* strain AG, have demonstrated the ability to directly debrominate polybrominated diphenyl ethers (PBDEs) under various growth conditions, including organic-carbon-free medium [37]. This finding expands the known capabilities of *Acetobacterium* in halogenated organic compound transformations.

CO is a naturally occurring compound in underground environments, potentially serving both as an electron donor and a carbon source for a variety of microorganisms. In this study, we hypothesize that CO could fuel reductive dechlorination within mixed microbial communities, specifically supporting the activities of OHRB (e.g., *Dehalococcoides*). To test this hypothesis, we established microcosms using river sediment as a microbial source, exploring the potential of CO as a supportive factor for sustained dechlorination. Our investigations revealed that CO, even at concentrations exceeding 2.2 µM—previously deemed detrimental to *Dehalococcoides* growth [27]—effectively promoted the survival, growth, and enrichment of the key dechlorinator. These revelations carry substantial implications, shedding light on the conceivable role of CO as both electron donor and carbon source for diverse microorganisms inhabiting subterranean environments, including the less-recognized OHRB. Additionally, they underscore the significance of CO in facilitating the reductive dechlorination activities of OHRB beyond the scope previously acknowledged.

## Materials and methods

### Chemicals

Trichloroethylene (TCE) and *cis*-1,2-dichloroethylene (*c*DCE) were purchased from Macklin Biochemical Co., Ltd (Shanghai, China). Vinyl chloride (VC) and ethene (both ≥ 99%) were purchased from Dalian Special Gases Co., Ltd (Dalian, China). H_2_, nitrogen (N_2_), carbon dioxide (CO_2_), and CO (all ≥ 99.999%) were purchased from Shuntai Special Gases Co., Ltd (Shenyang, China). All other chemicals used in this study were analytical grade or of higher purity.

### Microcosm setup and enrichment cultures

Medium preparation and anaerobic cultivation were performed following established protocols [39–41]. Briefly, a reduced, bicarbonate-buffered mineral salt medium was boiled under an atmosphere of N_2_ to remove dissolved oxygen, cooled down to room temperature, and then dispensed into serum bottles flushed with N_2_/CO_2_ (80/20, v/v). Sediment samples were collected from a location (41° 39′ 46″ N, 123° 6′ 20″ E) at Xi River, Shenyang, Liaoning Province, China, as described [42]. Microcosms were constructed inside an anoxic chamber (Coy Laboratory Inc., MI, USA) filled with N_2_/H_2_ (97/3, v/v). An aliquot of 2 mL sediment sludge was pipetted into 120-mL glass serum bottles prefilled with 80 mL of medium mentioned above as described [40, 43]. Bottles were sealed with butyl rubber stoppers (Fushiyuan rubber and plastic products factory, Shenzhen, Guangdong, China) and crimped with aluminum caps (Hongpu Instrument Technology, Ningbo, Zhejiang, China). Initially, acetate (5 mM) was provided as the carbon source, and CO (2 mL) was provided as the electron donor. In the incubation period, CO was added to several doses (2 mL each). Neat TCE (3 µL, *ca.* 0.3 mM or 43.4 mg/L aqueous phase concentration) was added as the electron acceptor. All bottles were amended with Wolin vitamin mix [44]. Following the complete dechlorination of TCE to ethene, 1 mL culture suspension was transferred into a fresh medium following the same procedures (Fig. S1). The bottles were incubated statically in the dark at 30 °C. Microcosms and enrichment cultures were established in triplicate bottles. Cultures amended without CO or with H_2_ as the electron donor served as controls.

### Sequencing, assembly, and binning

Metagenome sequencing was performed by Novogene Co., Ltd. (Beijing, China). DNA samples were extracted from the fourth transfer enrichment cultures with CO as both carbon source and electron donor using the CTAB protocol [45]. DNA degradation and potential contamination were monitored on 1% agarose gels. DNA concentration was measured using Qubit® dsDNA Assay Kit in Qubit® 2.0 Fluorometer (Life Technologies, CA, USA). Sequencing libraries were generated using NEBNext® Ultra™ DNA Library Prep Kit for Illumina (NEB, USA) following manufacturer’s recommendations, and index codes were added to attribute sequences to each sample. The clustering of the index-coded samples was performed on a cBot Cluster Generation System. After cluster generation, the library preparations were sequenced on an Illumina Hiseq platform, and paired-end reads were generated. Raw sequence data were processed with Readfq v8 (https://github.com/cjfields/readfq) to acquire the filtered sequence data for subsequent analysis. After being trimmed and filtered, the resulting 35,338,878 paired-end reads were assembled using the JGI Metagenome Assembly Pipeline (https://github.com/kbaseapps/jgi_mg_assembly) [46]. Metagenomic short-read profiling and taxonomic classification were performed using Kaiju v1.7.3 [47]. Metagenomic contigs were classified with Maxbin2 v2.2.4 [48]. The metagenome-assembled genomes (MAGs) were assessed with CheckM [49] using default settings for completeness and contamination evaluation. High-quality MAGs that included the draft genome sequence of a *Dehalococcoides* strain, designated as strain CO, were annotated using BV-BRC v3.29.20 (https://www.bv-brc.org/) and RAST v2.0 (https://rast.nmpdr.org/) with default parameters. The procedures for DNA extraction, amplicon sequencing, Sanger sequencing, PCR, and qPCR are elaborated in the Supplementary Information (SI).

### Analytical methods

Ethene, methane, and chlorinated compounds were analyzed using an Agilent 7890B gas chromatography (GC) equipped with an Agilent 7697A automatic headspace sampler (Agilent Technologies, Santa Clara, CA, USA), a flame ionization detector (FID) (method detection limit ~ 0.2 µM) and an Agilent DB-624 capillary column (60 m length × 0.32 mm inner diameter × 1.8 μm film thickness) as described [40]. Oven temperature was initially held at 60 °C for 2 min, increased to 200 °C at 25 °C/ min, and held at 200 °C for 1 min. Inlet and FID temperatures were set at 200 °C and 300 °C, respectively [42].

H_2_ and CO were analyzed using a Peak Performer 1 (PP1) 910-100 trace level gas chromatography equipped with a reducing compound photometer (RCP) (method detection limit ~ 1 ppb) (Peak Laboratories, CA, USA). Column and RCP bed temperatures were set at 105 °C and 265 °C, respectively.

Acetate was analyzed using an Agilent 1260 high-performance liquid chromatography (HPLC) system (Agilent Technologies, Santa Clara, CA, USA) equipped with an Aminex HPX-87H column (Bio-Rad, Hercules, CA, USA) and a diode array detector (DAD) set at 210 nm; samples were separated at a flow rate of 0.6 mL/min using 4 mM H_2_SO_4_ as the mobile phase [50].

### Data availability

The BioProject accession number is PRJNA1042952. The 16S rRNA gene amplicon sequencing data are available in the Sequence Read Archive of GenBank under the accession number SRR27024736 (CO, 5th transfer, HEPES buffer), SRR27024737 (CO, 5th transfer, bicarbonate buffer), SRR27024738 (CO, 3rd transfer, bicarbonate buffer), and SRR27024739 (CO and acetate, 3rd transfer, bicarbonate buffer). The binning genomic sequence of *Dehalococcoides* sp. strain CO is available in GenBank under the accession number JBDODF000000000. The binning genomic sequence of *Acetobacterium* sp. strain Z1 is available in GenBank under the accession number JBDODG000000000. Three *rdhA* genes (RdhA1, RdhA2, and RdhA3) annotated from the draft *Dehalococcoides* sp. strain CO are available in GenBank under the accession number PP060998, PP060999, and PP061000. The partial 16S rRNA gene sequence of *Dehalococcoides* sp. strain CO is available under the accession number OQ946896.

## Results

### CO as an electron source for reductive dechlorination of TCE

**Fig. 1:**
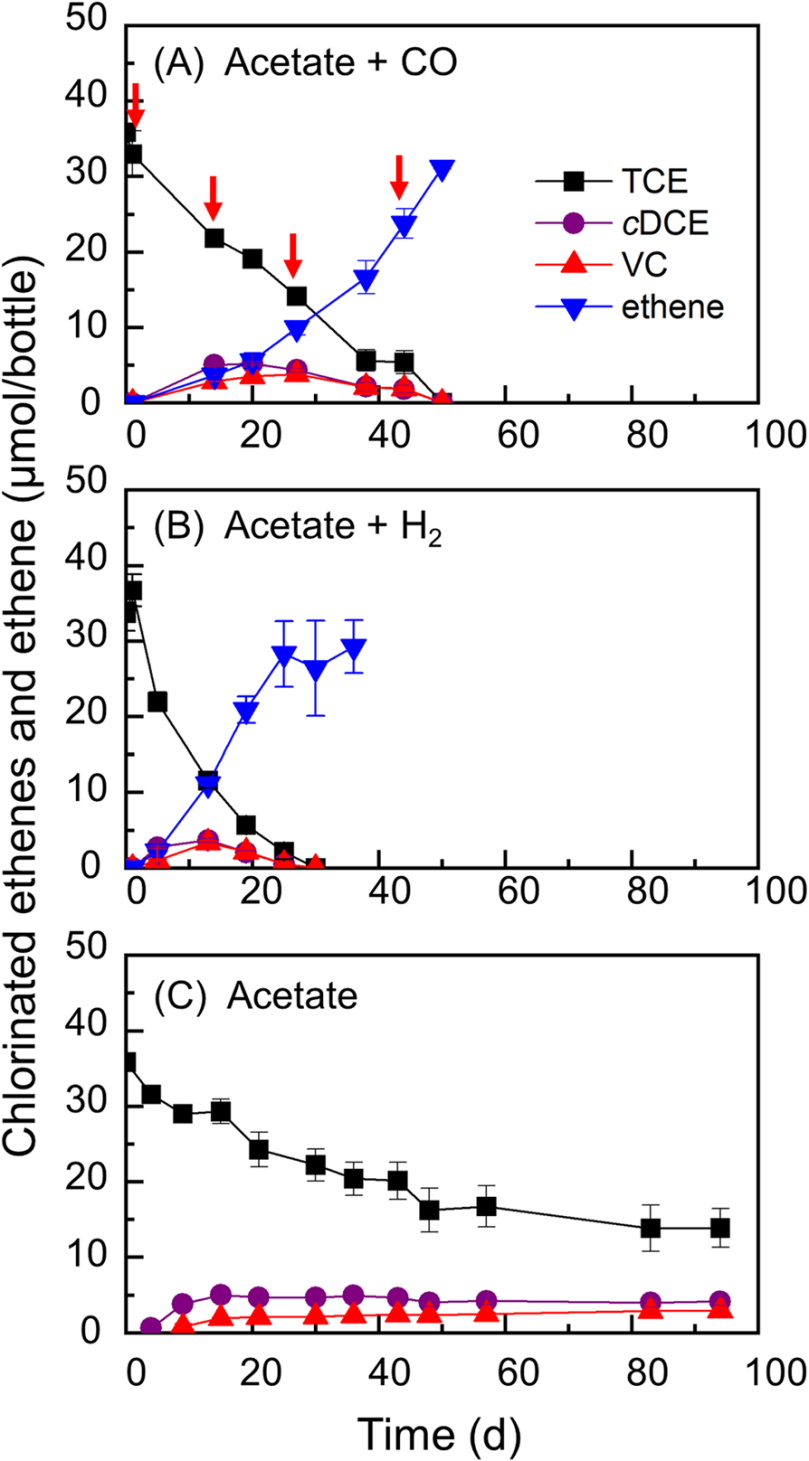
Reductive dechlorination of TCE in enrichment cultures amended with CO plus acetate. Hydrogenolysis of TCE to ethene using CO (**A**) or H_2_ (**B**) as the electron donor. Stalled dechlorination of TCE in the absence of an electron donor (**C**). Error bars represent the standard deviations of triplicate samples, omitted when smaller than the symbol. The red arrows indicate CO supplementation (2 mL/dose)

After a 3-month incubation period, the sediment microcosms supplemented with 5 mM acetate and 2 mL of CO completely dechlorinated the initial 33.9 ± 1.6 µmol of TCE to ethene. In contrast, microcosms supplemented only with 5 mM acetate and without CO were unable to achieve complete TCE dechlorination, with the process stalling at the *c*DCE stage with negligible amount of VC (data not shown). Over the incubation period, five doses of CO (i.e., 10 mL in total) were added into the bottles. This led to the reductive dechlorination of TCE to ethene in approximately 90 days, with *c*DCE and VC produced sequentially (Fig. S2A). Following four consecutive transfers, the culture maintained the ability of complete reductive dechlorination of TCE to ethene within ~ 50 days (Fig. 1A). Specifically, TCE was dechlorinated to ethene within 30 days when H_2_ was provided as the electron donor (Fig. 1B), while TCE dechlorination stalled in approximately 100 days without H_2_ or CO amendment (Fig. 1C).

### Exclusive utilization of CO to support TCE reductive dechlorination

**Fig. 2:**
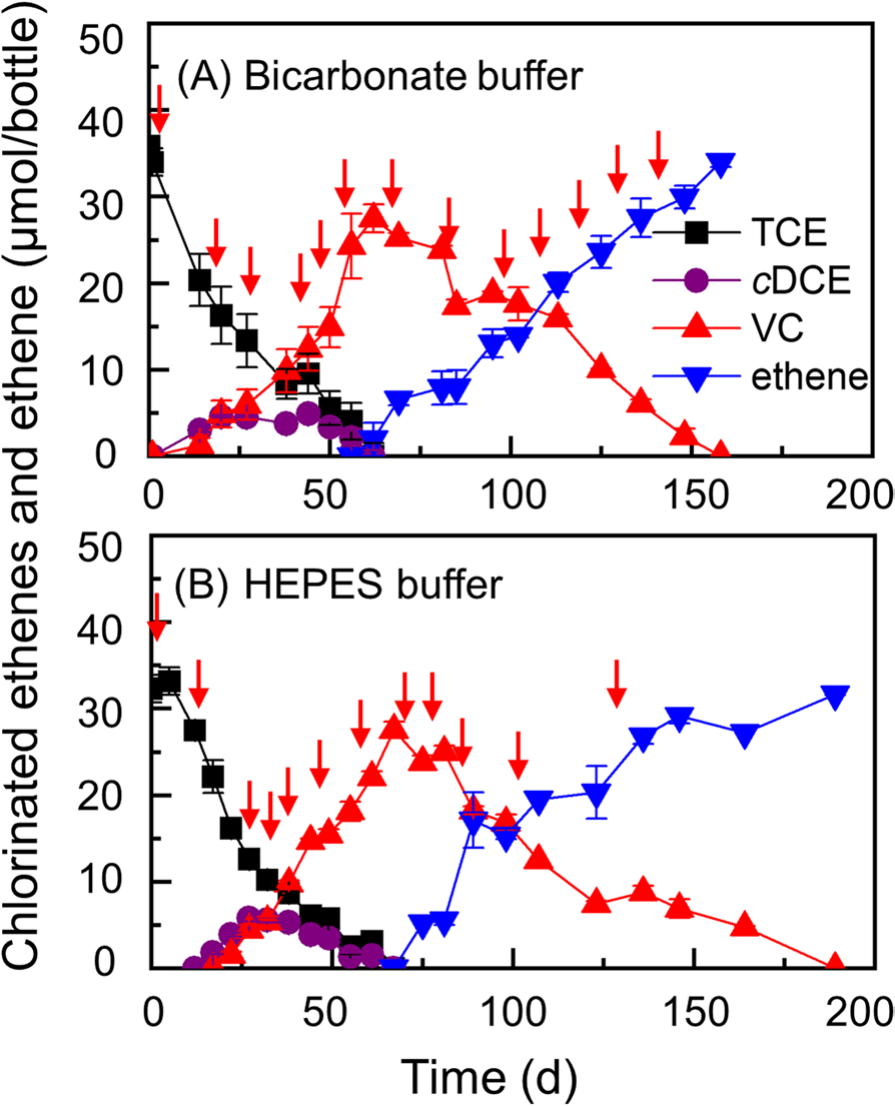
Reductive dechlorination of TCE to ethene by enrichment cultures only supplemented with CO in bicarbonate-buffered medium (**A**) or in HEPES-buffered medium (**B**). Error bars represent the standard deviations of triplicate samples, omitted when smaller than the symbol. The red arrows indicate the time points of CO supplementation (2 mL/dose)

Separate microcosms were established solely with CO as the hypothesized electron and carbon source (Fig. S1). After an extended incubation period of exceeding 6 months, the sediment microcosm amended with only 2 mL of CO unexpectedly achieved the dechlorination of the initial 32.5 ± 1.6 µmol of TCE to stoichiometric amounts of VC (data not shown), and the transferred cultures dechlorinated the same amount of TCE to ethene with increased dechlorination rates (Fig. S3). Subsequently, the fourth transfer, cultivated in the bicarbonate-buffered medium amended with thirteen doses of CO (i.e., 26 mL in total), demonstrated stepwise reductive dechlorination of TCE to ethene in approximately 160 days (Fig. 2A). To further substantiate the role of CO as the sole electron and carbon source, the bicarbonate buffer system was replaced with HEPES buffer, and the headspaces were purged with pure N_2_ gas. By comparison, the transferred cultures, cultivated in the HEPES-buffered medium and amended with only CO (~ 24 mL in total), also stepwise dechlorinated the same amount of TCE to 31.7 ± 0.1 µmol ethene in about 190 days, affirming that CO served not only as the electron donor but also as the carbon source (Fig. 2B).

### Microbial community profiles of TCE-dechlorinating enrichment cultures sustained by CO

**Fig. 3:**
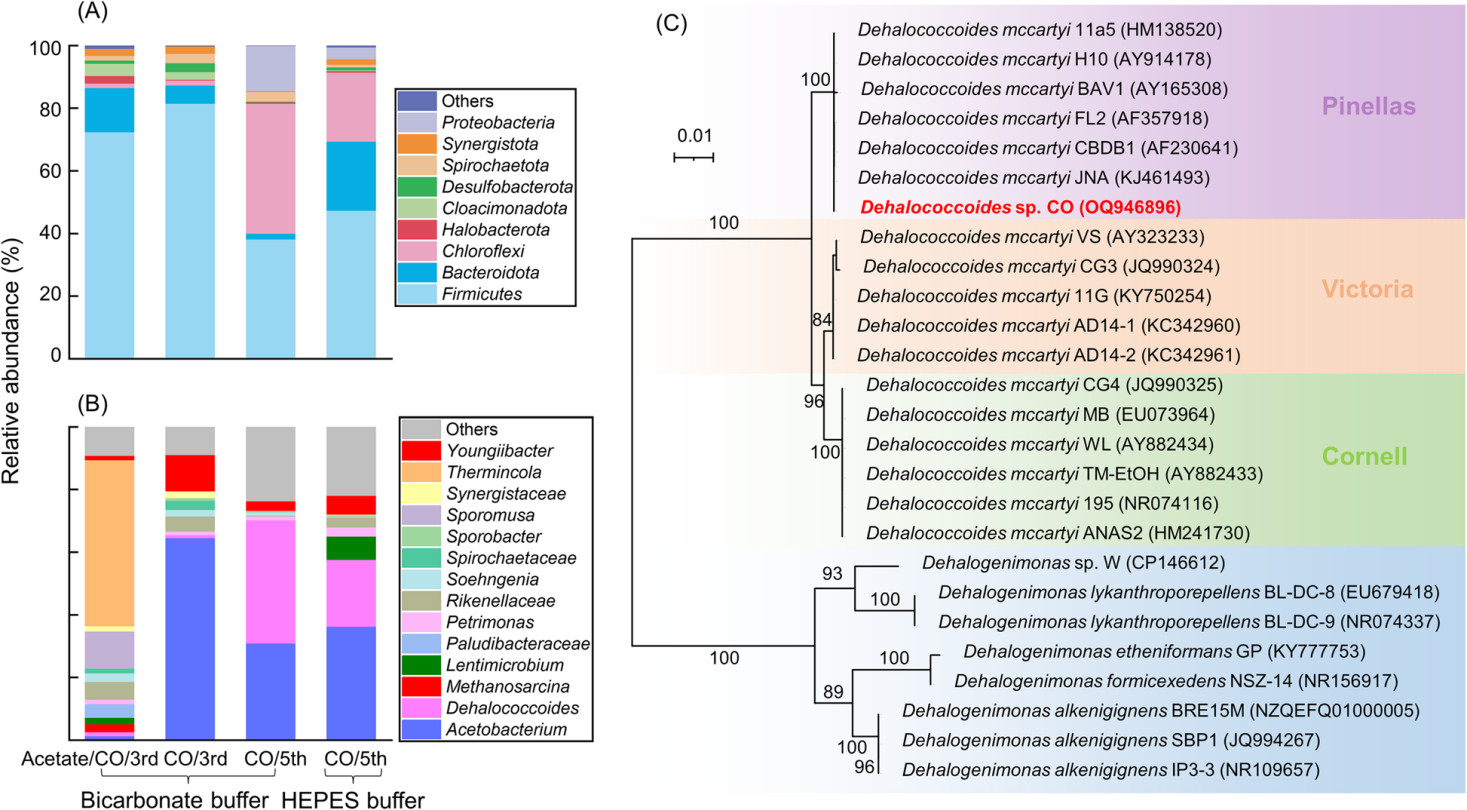
Microbial community structures at both the phylum (**A**) and genus (**B**) levels in the enrichment cultures following complete TCE depletion. Maximum-likelihood phylogenetic tree of *Dehalococcoides* (including its three subgroups) and *Dehalogenimonas* based on 16S rRNA gene sequences (**C**). Bootstrap values (1000 replicates) are indicated at branch points, and the scale bar represents nucleotide substitutions per site. GenBank accession numbers are provided in parentheses

Amplicon sequencing of the 16S rRNA gene was applied to investigate the microbial population(s) responsible for CO-fueled TCE dechlorination. The enrichment cultures cultivated under different conditions exhibited varying compositions at the phylum level, with *Firmicutes*, *Bacteroidota*, *Chloroflexi*, *Proteobacteria*, *Halobacterota*, and *Cloacimonadota* being the major phyla (Fig. 3A). Notably, the phylum *Chloroflexi* became dominant in enrichment cultures amended with CO as the electron donor and carbon source, regardless of the buffering agent used (bicarbonate or HEPES). At the genus level, *Thermincola* emerged as the predominant bacteria (53.0%) in enrichment cultures supplemented with both CO and acetate. Nonetheless, its presence was entirely absent in cultures lacking acetate amendment. This observation aligns with the known physiological capabilities of *Thermincola carboxydiphila* strain 2204, a described alkalitolerant, CO-utilizing, H_2_-producing, thermophilic anaerobe [51], which possesses the ability for chemolithotrophic growth via anaerobic CO oxidation coupled to H_2_ and CO_2_ production [51, 52]. The second most abundant genus in CO plus acetate enrichment cultures was *Sporomusa* (11.9%), capable of growth on either CO or H_2_/CO_2_ [53]. Methanogenic archaea *Methanosarcina* (2.5%) was exclusively detected in CO plus acetate enrichment cultures, with ~ 450 µmol/bottle of methane detected. In the third transferred cultures amended with CO only, the most abundant genus was *Acetobacterium* (64.6%), whereas its relative abundance in the third transfer cultures amended with CO plus acetate was only 1.3%. Given the capacity of several *Acetobacterium* species to convert CO to acetate [54], it is hypothesized that *Acetobacterium* in the CO-fueled enrichment cultures serves as the primary producer and provides carbon source acetate for other populations within the community. It is worth noting that the model acetogen *Acetobacterium woodii* cannot grow on CO as a sole carbon and energy source, suggesting that the *Acetobacterium* identified in this study may differ from *Acetobacterium woodii* [10]. Meanwhile, *Acetobacterium wieringae* strain JM has the ability to grow with CO as both carbon and energy source and was isolated recently [55]. *Dehalococcoides* (1.1–39.3%) was the only OHRB phylotype detected in all the enrichment cultures. *Acetobacterium* and *Dehalococcoides* were the top two most abundant genera in the fifth transfers cultivated with CO only (Fig. 3B). Subsequently, we obtained a nearly complete (i.e., ~ 1300 bp) 16S rRNA gene from the CO-fed consortium using the *Dehalococcoides*-specific primers. As shown in Fig. 3C, the amplicon shared 98.5–100% sequence similarities with the 16S rRNA gene of known *Dehalococcoides* isolates (e.g., 195, BAV1), which provides additional evidence for the presence of a *Dehalococcoides* population in the CO-fed TCE-dechlorinating community. We hypothesized that certain genera employ CO as a precursor to generate CO_2_ and H_2_. *Acetobacterium*, in turn, harnesses these products to synthesize acetate. The symbiotic interaction involving H_2_ and acetate facilitates the survival of *Dehalococcoides* and enhances its capability to reductively dechlorinate TCE to ethene within the microbial community.

### *Dehalococcoides* growth coupled with TCE reductive dechlorination fueled by CO

**Fig. 4:**
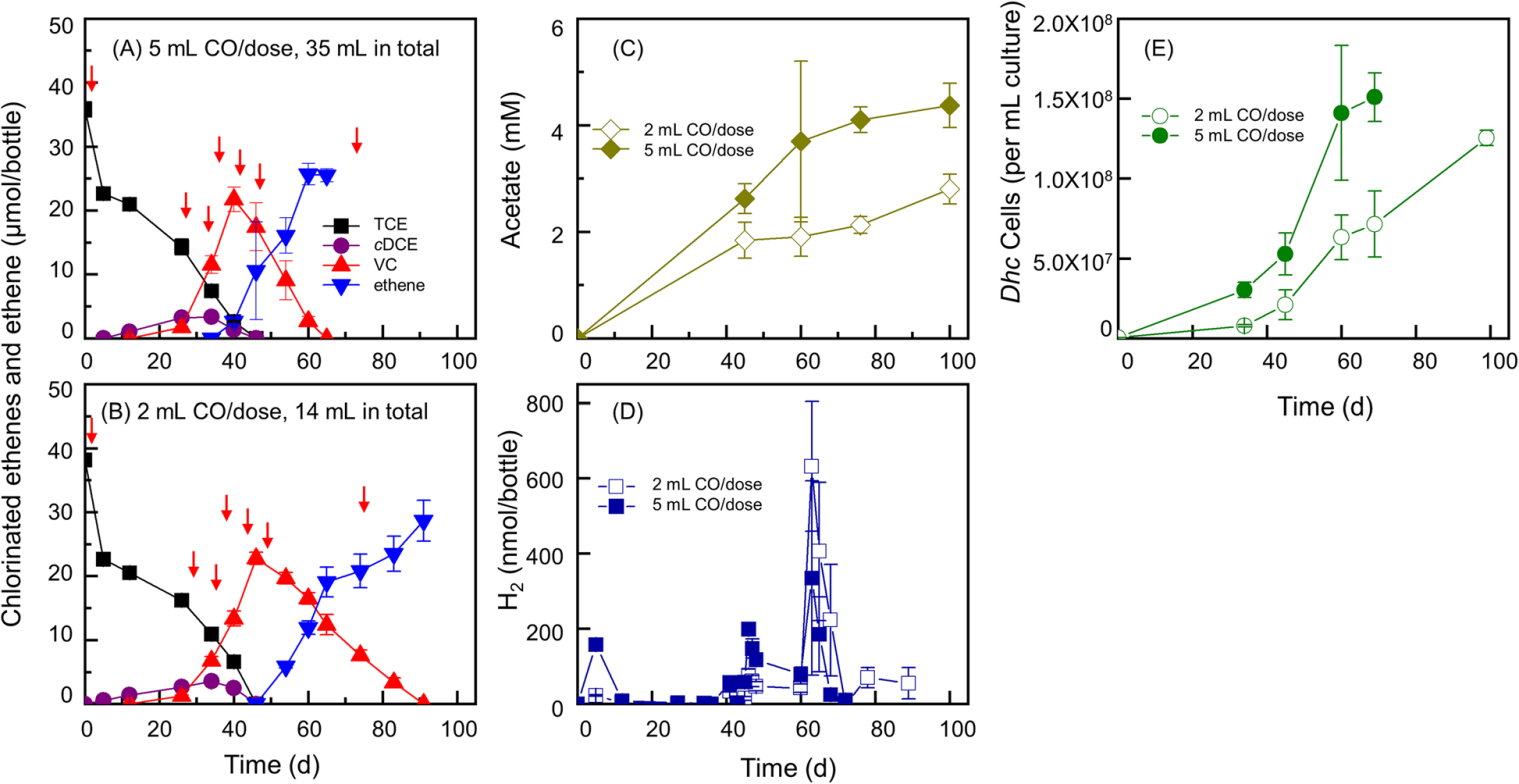
Reductive dechlorination of TCE by enrichment cultures exclusively amended with 5 mL CO/dose (**A**) and 2 mL CO/dose (**B**) coupled with acetate formation (**C**), H_2_ formation (**D**), and *Dehalococcoides* growth (**E**). Concurrently, the figures detail the growth of *Dehalococcoides* (**C**), the formation of H_2_ (**D**), and the production of acetate (**E**). Error bars, reflecting standard deviations from triplicate samples, are omitted when their magnitude is below the symbol. The red arrows in the figures indicate the specific points of CO supplementation

To elucidate the crucial role of CO in facilitating *Dehalococcoides* growth and TCE dechlorination, we conducted an experiment involving the transfer of CO enrichment cultures (in a bicarbonate buffer) with varying CO supplement doses, resulting in total added CO of 14 mL (571.4 µmol) or 35 mL (1428.6 µmol), respectively. Our findings revealed that the initial 38.1 ± 0.3 µmol TCE was dechlorinated to stoichiometric amounts of ethene within approximately 90 and 65 days in cultures supplemented with 2 mL CO/dose and 5 mL CO/dose, respectively. A clear correlation was observed between the amount and frequency of CO supplementation and the rate of TCE dechlorination (Fig. 4A, B). Concomitant with TCE dechlorination, *Dehalococcoides* cell numbers in the cultures increased significantly from (3.6 ± 0.3) × 10^5^ to (1.5 ± 0.2) × 10^8^ cells per mL cultures (416.9-fold increase) and (1.3 ± 0.1) × 10^8^ cells per mL cultures (346.4-fold increase), respectively (Fig. 4E). Concomitant with the supplementation of CO, a noteworthy increase in H_2_ production was observed. However, despite significant CO additions, H_2_ generation during the TCE-to-VC dechlorination phase remained exceptionally low (maximum amounts measured were 33.7 ± 1.4 nmol/bottle and 158.1 ± 6.9 nmol/bottle for 2 and 5 mL CO doses, respectively) over approximately 40 days. Intriguingly, the accumulation of H_2_ was exclusively evident during the VC-to-ethene transition, with the maximum amounts measured at 631.8 ± 172.5 nmol/bottle and 334.7 ± 158.2 nmol/bottle, respectively (Fig. 4D). This stage-specific pattern suggests a potential shift in metabolic pathways or microbial community dynamics during the dechlorination process. Additionally, CO-fueled acetogenesis by *Acetobacterium* was evident in the enrichment cultures, as depicted by the final acetate concentrations of 2.4 ± 0.01 mM and 4.4 ± 0.4 mM in the 2 mL and 5 mL CO/dose cultures, respectively (Fig. 4C). This acetate production potentially serves as a substrate for other microbial populations within the community, further influencing the observed H_2_ dynamics.

### Draft genome of the TCE-dechlorinating *Dehalococcoides*

The binning of metagenomic contigs resulted in the assembly of a draft *Dehalococcoides* genome, designated as strain CO. This genome comprised 4 contigs with a total size of 1,360,741 bp, a G + C content of 47.2% and N50 = 781,482 bp. CheckM analysis indicated that the genome was nearly 95.1% complete with 1.5% contamination [49]. PATRIC and RAST annotation of the draft genome predicted a total of 1497 genes including 1440 coding sequences (CDS) and 57 non-coding RNA sequences. *Dehalococcoides* strain CO exhibited a high genome-aggregate average nucleotide identity (ANI) with strains CBDB1 (99.3%), FL2 (99.7%), 11a5 (99.4%), and KS (99.4%) (Table S1), exceeding the 95% ANI threshold for species demarcation [56]. However, strain CO showed lower ANI compared to strains 195 (86.3%), VS (86.8%), and CG3 (87.7%). A total of 20 *rdhA* were annotated in the draft genome of strain CO. Two identical RDases RdhA1 (NCBI Accession #PP060998) and RdhA2 (NCBI Accession #PP060999) were annotated in the draft genome of strain CO, sharing amino acid identities of 96.5%, 96.9%, 97.9%, and 96.7% with the VcrA protein sequences in the KB-1 consortium, *Dehalococcoides* strain VS, strain WBC-2, strain GT, respectively (Fig. S3). Another RDase RdhA3(NCBI Accession #PP061000) with a full length of 500 amino acids shared 94.4% and 99.6% amino acid identities when compared with TceA in *Dehalococcoides* strain 195 and the KB-1 consortium, respectively (Fig. S4). Other putative RDases exhibited relatively lower similarities to the charactered RDases.

### Draft genomes of the CO-oxidating anaerobes

Binning of the metagenomic contigs enabled the assembly of a draft *Acetobacterium* genome, designated as strain Z1. This genome consisted of 46 contigs with a total size of 3,373,213 bp, a G + C content of 44.5%, and N50 = 781,482 bp. CheckM analysis indicated that the genome was almost 100% complete with 0.3% contamination [49]. PATRIC and RAST annotation of the draft genome predicted a total of 3298 genes, including 3255 coding sequences (CDS) and 43 non-coding RNA sequences. Strain Z1 exhibited relatively lower ANI (< 90%) and dDDH (< 70%) compared to other *Acetobacterium* species (Table S3). The comparison and phylogenetic tree based on the genomes (Fig. S5) indicated that strain Z1 represented a new species within the genus *Acetobacterium*. No RDase genes were annotated in the draft genome of strain Z1, suggesting it is not capable of organohalide respiration. The CODH annotated in the draft genome of strain Z1 shared 97.7%, 95.3%, 94.8%, 92.7%, and 90.0% amino acid identities with the CODH protein sequences in *Acetobacterium woodii*, *Acetobacterium malicum*, *Acetobacterium wieringae*, *Acetobacterium dehalogenans*, and *Acetobacterium tundrae*, respectively (Fig. S6). Enrichment cultures harbored a diverse microbial community, with several prominent genera—*Acetobacterium*, *Methanosarcina*, *Desulfomicrobium*, and *Desulfocurvibacter*—exhibiting the genomic potential for CO utilization via the presence of CODH. The draft genome of strain Z1 encompasses the complete WLP, with the key enzyme ACS likely playing a crucial role in survival and growth within the CO-fed cultures. H_2_ formation from CO oxidation serves as a central reaction, driving electron flow throughout the community. This highlights the increasing recognition of hydrogenogenic carboxydotrophs, identified in diverse environments [57]. While H_2_ production by *Acetobacterium* remains unreported, *Acetobacterium wieringae* and *Acetobacterium woodii* are known to generate CO_2_ and acetate as CO oxidation end products [55, 58]. The genes encoding the CO oxidation system (Coo) and energy-converting hydrogenase (Ech) serve as marker genes for hydrogenogenic carboxydotrophy [59, 60]. Genomic annotations revealed that *Desulfomicrobium*, *Desulfocurvibacter*, *Methanosarcina*, *Methanoculleus*, *Methanosarcina*, and *Methanofollis* were annotated with both Coo and Ech, establishing them as potential H_2_ producers in the CO-fed cultures.

## Discussion

To date, the complete detoxification of chlorinated ethenes into the environmentally benign product ethene relies exclusively on a specific subset of OHRB within the class *Dehalococcoidia* (e.g., *Dehalococcoides*, *Dehalogenimonas*) [17, 40]. It is noteworthy that all *Dehalococcoides* isolates exhibit a strict requirement for H_2_ as electron donor, an indispensable role that cannot be substituted [17]. Various pathways for H_2_ production have been demonstrated, including organic acids fermentation, phosphite oxidation, acetate oxidation, nitrogen fixation, and anaerobic carbon monoxide oxidation [61]. The established knowledge regarding the evolution of H_2_ supporting OHRB has been well documented. Here, we demonstrate that CO can serve as an alternative electron donor for the dechlorination of chlorinated ethenes, ultimately yielding the environmentally friendly end product ethene.

A total of 1428.6 µmol of CO (i.e., 35 mL CO) were meticulously introduced into the 5 mL CO/dose supplemented enrichment cultures. Following the principles delineated in Table 1, it can be deduced that each molecule of CO can be converted into one molecule of H_2_, as depicted by equation 2. Thus, the maximum theoretical H_2_ production would amount to 1428.6 µmol. In accordance with equation 1, the complete dechlorination of 33.3 µmol of TCE to ethene would theoretically consume 100.0 µmoles of H_2_. The remaining H_2_, if exclusively utilized for acetate production, would reach a substantial quantity of 332.1 µmol, equivalent to 4.2 mM in an 80 mL medium, as indicated by equation 3. Remarkably, the measured final concentration of acetate was determined to be 4.4 ± 0.4 mM, underscoring the intricate metabolic dynamics orchestrated within the microbial community.

**Table 1:** Gibbs standard-state free energy changes for redox reactions in the system

**Reactions**	**Equations**^**a**^		**ΔrG′° (kJ/mol)**^**b**^
TCE dechlorination	C_2_Cl_3_H + 3 H_2_ (g) → C_2_H_4_ (g) + 3 HCl	(1)	−150.5
H_2_ formation	CO (g) + H_2_O → CO_2_ (g) + H_2_ (g)	(2)	−20.1
Acetogenesis	2 CO_2_ (g) + 4 H_2_ (g) → 2 H_2_O + CH_3_COOH	(3)	−75.3
Acetogenesis^c^	4 CO (g) + 2 H_2_O → 2 CO_2_ (g) + CH_3_COOH	(4)	−155.7
Methanogenesis	CH_3_COOH → CO_2_ (g) + CH_4_ (g)	(5)	−55.0
Methanogenesis	CO_2_ (g) + 4 H_2_ (g) → 2 H_2_O + CH_4_ (g)	(6)	−130.3
Methanogenesis	4 CO (g) + 2 H_2_O → 3 CO_2_ (g) + CH_4_ (g)	(7)	−210.7

^a^H_2_, CO, CO_2_, and ethene are treated as gaseous species (g) for Gibbs free energy calculation

^b^Calculated under standard conditions (i.e., temperature of 298.15 K [25 °C], pH of 7.0, concentrations of solutes at 1 M and partial pressures of gases at 1 atmosphere), data source of the Gibbs standard-state free energy of the compounds were CRC Handbook of Chemistry and Physics, 84th Edition (2004) [62]

^c^WLP with CO as carbon and energy source

The analyses of carbon and electron balance revealed that the principal outcomes of CO oxidation in our study were the production of acetate, CO_2_, and H_2_ (Table 2). These findings underscored the intricate interplays among CO oxidation, TCE dechlorination, and WLP in the metabolic processes occurring within the enrichment cultures. Within the enrichment cultures, a remarkable division of labor emerged concerning CO utilization. *Acetobacterium* assumed the primary role in converting CO into acetate, likely via the WLP. Concurrently, several hydrogenogenic carboxydotrophs (e.g., *Desulfomicrobium*, *Desulfocurvibacter*, *Methanosarcina*) potentially drove the alternative pathway, transforming CO into CO_2_ and H_2_. This strategic cooperation provided a dual benefit: H_2_ and acetate, generated by the initial CO oxidation, were subsequently channeled to *Dehalococcoides* for fueling its reductive dechlorination of TCE. CO functioned as an indirect yet crucial source of both energy and carbon for *Dehalococcoides*, enabling its vital role in the overall dechlorination process.

**Table 2:** Carbon and electron balance in CO-fed enrichment

Treatments	Initial substrate (µmols/bottle)		Final products (µmols/bottle)			Carbon recovery^a^ (%)	Electron recovery^b^ (%)
	CO	TCE	Methane	Ethene	Acetate		
2 mL/dose CO	571.4	38.2	3.2	28.7	170.1	61.9	116.4
5 mL/dose CO	1428.6	35.8	11.0	25.5	349.6	50.7	100.4

^a^The carbon and electron balance calculations were based on the utilized substrates and the formation of methane, ethene, and acetate, excluding CO_2_. The low carbon recovery observed in the balance calculations is attributed to the exclusion of CO_2_ from the analysis

^b^The electron balance calculations were based on the effective electron number of elements C, H, and O, which are 4, 1, and −2, respectively

Previous research had indicated the detrimental effect of CO, inhibiting the reductive dechlorination of TCE and hexachlorobenzene driven by *Dehalococcoides* strain 195 or CBDB1, respectively [27, 63]. Even a modest concentration of 6 µmol per bottle for strain 195 or 1 µmol per bottle for strain CBDB1 of CO could severely impede the growth of *Dehalococcoides* [27]. Additionally, CO accumulation as a metabolic by-product in dechlorinating cultures dominated by *Dehalogenimonas etheniformans* strain GP has been shown to negatively impact reductive dechlorination activity. Externally amended CO at 4 µmol (~ 880 ppmv in the culture vessel) strongly inhibited vinyl chloride (VC) degradation by strain GP, indicating *Dehalogenimonas* strains, like *Dehalococcoides*, are sensitive to CO. These findings underscore the need for strategies (e.g., syntrophy) to mitigate CO toxicity in dechlorinating systems comprising obligate OHRB like *Dehalococcoides* and *Dehalogenimonas* [64, 65]. Surprisingly, in our study, we amended a maximum CO (5 mL) concentration of up to 204.1 µmol per bottle. Contrary to expectations, not only did *Dehalococcoides* endure under such elevated CO concentrations, but it also thrived and proliferated by harnessing CO, a specific interaction mechanism hitherto undocumented in the literature. It is important to note that due to the dynamic nature of CO dissolution and consumption in the liquid phase, the actual CO concentration experienced by *Dehalococcoides* in our enrichment culture was likely substantially lower than the calculated equilibrium concentration. Therefore, the true CO tolerance of *Dehalococcoides* in our system could be lower than the levels supplemented in the culture vessels.

While CO offers advantages in terms of energy conservation compared to H_2_, its utilization by microorganisms has been restricted by issues related to microbial tolerance [8]. Nevertheless, certain anaerobes have demonstrated the capacity to utilize CO for the production of carboxylates and alcohols. Other than that, the coupling of CO oxidation with various respiratory processes, such as desulfurication, hydrogenesis, acetogenesis, and methanogenesis, has been established [58]. For instance, *Clostridium ljungdahlii* can produce acetate and ethanol through WLP using CO [66]. Methanogens, especially *Methanosarcina acetivorans*, have been extensively studied for their ability to grow on CO as the sole substrate, with the concomitant formation of acetate [58, 67]. However, only a limited number of anaerobes capable of utilizing CO as their sole source of energy and carbon have been documented to date. *Acetobacterium*, a well-studied anaerobic microorganism possessing a complete WLP, is also known for its ability to utilize CO. However, the capacity to grow solely on CO as the carbon and energy source has only been observed in one strain, JM, to date [10, 55, 68]. In our study, we demonstrated that CO could effectively function as the sole carbon and energy source, thereby maintaining the stability of the microbial community. *Acetobacterium* spp. are frequently co-cultured with OHRB, playing a significant role in their activities: (1) they provide essential metabolites like acetate, vitamin B_12_, and other cofactors to support the growth and dehalogenation capabilities of OHRB such as *Dehalococcoides*, *Trichlorobacter* (formerly *Geobacter*), and *Sulfurospirillum*; (2) they can mitigate the toxicity of CO, a common inhibitor of OHRB; and (3) certain strains, like *Acetobacterium* strain AG, possess the ability to directly debrominate polybrominated diphenyl ethers (PBDEs), suggesting facultative organohalide respiration within the genus [35–38, 69–72]. Additionally, our findings regarding CO-dependent H_2_ production hold significant implications, offering a promising alternative in the context of diminishing fossil fuel resources [58]. It is crucial to acknowledge that H_2_ production coupled with CO oxidation has been infrequently observed, possibly due to technological limitations. The exploration of CO-dependent energy conservation presents an exciting avenue for future research.

H_2_ is produced in anoxic environments through the oxidation of organic matter [73, 74]. In addition to anaerobic fermentation, H_2_ can also be generated directly or indirectly through bio-photolysis, photo-fermentation, CO gas-fermentation, and nitrogen fixation [61, 75, 76]. For instance, nitrogen fixation, despite its energy consumption, results in the annual production of 2.4–4.9 Tg H_2_ per year [75]. These H_2_ can be rapidly consumed in microbial-mediated terminal electron-accepting processes, such as iron reduction, sulfate reduction, denitrification, methanogenesis, and organohalide respiration [73, 74]. By comparison, the organohalide respiration process can compete with other electron-accepting processes, possessing a thermodynamic advantage. This advantage arises from the ability of organohalide-respiring bacteria to utilize a relatively low threshold H_2_ concentration [77]. The H_2_ threshold concentrations for the reduction of various chlorinated compounds differ. For instance, the mean H_2_ concentrations during the reductive dehalogenation of 2,4-dichlorophenol (2,4-CP), 2,3,4-trichlorophenol (2,3,4-CP), pentachlorophenol (PCP), and tetrachloroethene (PCE) were 3.6 nM, 4.1 nM, 0.3 nM, and 0.8 nM, respectively [77]. H_2_ threshold concentrations range from 0.6 to 0.9 nM for PCE and TCE reduction, 0.1–2.5 nM for *c*DCE reduction, and 2–24 nM for VC reduction [78, 79]. In our study, a noticeable production of H_2_ from CO oxidation was observed only between approximately day 60 and day 75. We speculate that during the reductive dechlorination process from TCE to VC, H_2_ produced from CO oxidation was promptly utilized for dechlorination. Low concentrations of H_2_ may have failed to fuel VC dechlorination, resulting in H_2_ accumulation (Fig. 4D). These findings align with previous reports indicating that electron donor (e.g., H_2_) limitation can inhibit the growth of VC-dechlorinating *Dehalococcoides* populations [80]. The dynamic interplay between hydrogenogenic carboxydotrophy and *Dehalococcoides*, involving H_2_ transfer, indicates a microbial ecological collaboration with advantages. Further investigation is warranted to confirm the underlying mechanisms governing this intricate microbial interaction.

**Fig. 5:**
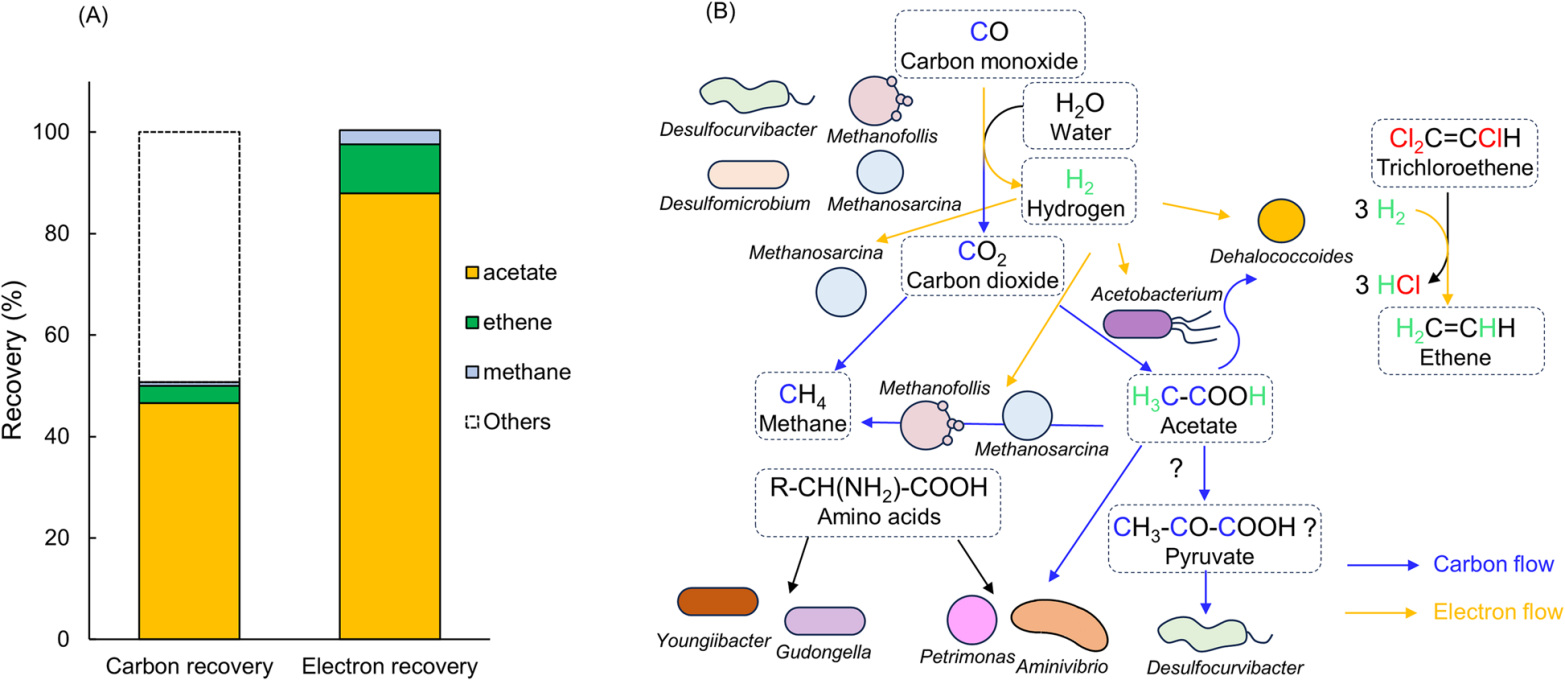
Carbon recovery and electron recovery, acetate, ethene, and methane were the main three products (**A**). Proposed interaction networks within CO-fed TCE-dechlorinating cultures based on assembled draft genomes of various genera suggest diverse metabolic pathways. CO oxidation to CO_2_ and H_2_ is hypothesized to occur in *Desulfocurvibacter*, *Desulfomicrobium*, and *Methanosarcina*, facilitated by the presence of CODH and Ech. *Acetobacterium* is identified as capable of exclusively oxidizing CO to acetate, attributed to the presence of complete WLP genes. Acetate and H_2_ collectively support the reductive dechlorination of TCE to ethene by *Dehalococcoides*. *Methanosarcina* and *Methanofollis* utilize acetate, H_2_, and CO_2_ to produce methane. Genera such as *Youngiibacter*, *Gudongella*, and *Petrimonas*, identified as fermentation specialists, demonstrate the ability to metabolize carbohydrates and some organic acids, while *Aminivibrio*, a fermentation bacterium, exhibits a preference for acetate utilization. These proposed interactions outline a complex web of metabolic relationships in the CO-enriched TCE-dechlorinating cultures (**B**)

In this study, in addition to *Dehalococcoides* and *Acetobacterium*, draft genomes for several other genera, including *Youngiibacter*, *Desulfocurvibacter*, *Gudongella*, *Methanofollis*, *Aminivibrio*, and *Petrimonas* (Table S4), were successfully assembled. *Youngiibacter*, a strictly anaerobic microorganism, ferments various carbohydrates into ethanol, formate, acetate, and CO_2_ [81]. It likely engages in the fermentation of unidentified carbohydrates in CO-supplemented enrichment cultures. *Desulfocurvibacter*, a sulfate-reducing bacterium, typically thrives through pyruvate fermentation [82]; by comparison, it may function as a CO consumer in this study. *Gudongella* strain W6^T^ exhibits N_2_-fixing capability and utilizes amino acids while refraining from growth on acetate [83]. *Methanofollis*, a strictly anaerobic archaeon, oxidizes CO to produce H_2_ and CO_2_, and employs H_2_ and acetate to produce methane in CO-fed cultures [84]. *Aminivibrio* and *Petrimonas* are anaerobic fermentative bacteria with the capacity to ferment organic acids [85, 86]. Nevertheless, elucidating the precise functions of these anaerobes within CO-oxidizing and dechlorinating microbial communities remains challenging and speculative at this juncture (Fig. 5). Top-down approaches, involving the reduction of microbial community complexity through serial dilution or the isolation of specific microorganisms, and bottom-up strategies, integrating and synthesizing co-cultures or tri-cultures, hold promise for providing insights into the potential ecological functions of these anaerobes.

In summary, the biologically mediated “water-gas shift reaction” (CO + H_2_O ⇌ CO_2_ + H_2_) is predominantly catalyzed by CODH in potential hydrogenogenic carboxydotrophy *Desulfomicrobium*, *Desulfocurvibacter*, *Methanosarcina*, *Methanoculleus*, *Methanosarcina*, and *Methanofollis*. This enzymatic process results in the formation of H_2_ and CO_2_, which are subsequently utilized by *Acetobacterium* and *Methanosarcina* in acetogenesis and methanogenesis, respectively. The produced acetate and H_2_ are accessible for *Dehalococcoides*, enabling the reductive dechlorination of TCE to ethene. Additionally, acetate serves as a versatile carbon source, potentially harnessed by *Aminivibrio*, *Petrimonas*, *Methanofollis*, *Methanosarcina*, and other microbial groups. H_2_, acting as a ubiquitous energy currency in anoxic environments, underscores its essential role in interspecies H_2_ transfer (IHT), crucial for maintaining the community structure and function of CO-fed enrichment cultures (Fig. 5).

### Supplementary Information

Additional file 1: Figure S1. Microcosm setup and transferred enrichment cultures. Trichloroethylene (TCE) was dechlorinated in enrichment cultures fed with acetate and CO (represented by the royal blue bottles). Similarly, TCE dechlorination occurred in CO-fed enrichment cultures with bicarbonate-buffered medium (represented by the green bottles) and HEPES-buffered medium (represented by the orange bottles). Conversely, in acetate-fed enrichment cultures without CO or H_2_ supplementation (represented by the yellow bottles), the TCE dechlorination process did not occur, denoted by the symbol “X”, signifying the inability to dechlorinate. Figure S2. Reductive dechlorination of TCE in CO plus acetate enrichment cultures from transfer 1 to transfer 3. Approximately 33 μmol of TCE was dechlorinated to ethene over 100 days in transfer 1 cultures fed with acetate and CO (A). In transfer 2 cultures, 33 μmol of TCE was dechlorinated to ethene within 30 days, and an additional 33 μmol of TCE was dechlorinated within 25 days (B). In transfer 3 cultures, 33 μmol of TCE was dechlorinated to ethene over 50 days (C). Red arrows indicate CO additions, with each dose amounting to 2 mL. Figure S3. Reductive dechlorination of TCE in CO-fed enrichment cultures from transfer 2 to transfer 4. Approximately 33 μmol of TCE was dechlorinated to VC over 120 days in transfer 2 cultures (A). In transfer 3 cultures, 33 μmol of TCE was dechlorinated to VC with a small amount of ethene in 80 days (B). In transfer 4 cultures, 33 μmol of TCE was dechlorinated to ethene over 160 days (C). Red arrows indicate CO additions, with each dose amounting to 2 mL. Figure S4. Phylogenetic tree constructed based on the amino acid sequences of 43 RDases, with branches calculated from 500 bootstrap iterations. Functional assignments of these RDases were determined through biochemical characterization, expression analysis, or phylogenetic inference, representing a diverse array of OHRB. The tree also includes 20 RDases annotated from the draft genome of strain CO, highlighted in blue branches. Figure S5. Phylogeny of *Acetobacterium* based on genome sequences. The tree was constructed using the maximum-likelihood method, with GenBank accession numbers provided in parentheses. Bootstrap values, derived from 1,000 resamplings, are indicated at branching points. Figure S6. Phylogenetic tree based on protein sequences of 63 carbon monoxide dehydrogenases (CODHs) from diverse bacteria and methanogens (archaea). CODHs from methanogens are indicated with a blue background, those from Acetobacterium with a green background, and CODHs identified in CO enrichment cultures are highlighted in blue. Figure S7. Proposed model for interspecies interactions supporting TCE-to-ethene dechlorination by *Dehalococcoides* with CO as electron donor and carbon source. Hydrogenogenic carboxydotrophs (e.g., *Methanosarcina*) oxidize CO, generating H_2_. This H_2_ is then transferred to *Acetobacterium*, which in turn produces acetate. Both acetate and H_2_ are subsequently utilized by *Dehalococcoides* for reductive dechlorination of TCE to ethene. Additionally, *Methanosarcina* can use acetate and H_2_ to produce methane. Table S1. The average nucleotide identity (%) and digital DNA-DNA hybridization (%) of strain CO with other strains in the genus of *Dehalococcoides*. Table S2. RDases with assigned functions and their host OHRB used in Figure S4. Table S3. The average nucleotide identity (%) and digital DNA-DNA hybridization (%) of strain Z1 with other strains in the genus of *Acetobacterium*. Table S4 Metagenome-assembled genomes recovered from CO-fed TCE-dechlorinating enrichment cultures.

## Data Availability

All sequencing data generated and analyzed in this study have been deposited in the NCBI Sequence Read Archive (SRA) under the accession numbers provided in the Data availability section. Additional data or materials relevant to the study are available from the corresponding author upon reasonable request.
