# Supplementary material for: CO-driven electron and carbon flux fuels synergistic microbial reductive dechlorination
Source: Microbiome. 2024 Aug 20;12:154. doi: 10.1186/s40168-024-01869-y (PMC11334346; doi:10.1186/s40168-024-01869-y)
Supplement: Supplementary file 2 — Additional file 1: Figure S1. Microcosm setup and transferred enrichment cultures. Trichloroethylene (TCE) was dechlorinated in enrichment cultures fed with acetate and CO (represented by the royal blue bottles). Similarly, TCE dechlorination occurred in CO-fed enrichment cultures with bicarbonate-buffered medium (represented by the green bottles) and HEPES-buffered medium (represented by the orange bottles). Conversely, in acetate-fed enrichment cultures without CO or H2 supplementation (represented by the yellow bottles), the TCE dechlorination process did not occur, denoted by the symbol “X”, signifying the inability to dechlorinate. Figure S2. Reductive dechlorination of TCE in CO plus acetate enrichment cultures from transfer 1 to transfer 3. Approximately 33 μmol of TCE was dechlorinated to ethene over 100 days in transfer 1 cultures fed with acetate and CO (A). In transfer 2 cultures, 33 μmol of TCE was dechlorinated to ethene within 30 days, and an additional 33 μmol of TCE was dechlorinated within 25 days (B). In transfer 3 cultures, 33 μmol of TCE was dechlorinated to ethene over 50 days (C). Red arrows indicate CO additions, with each dose amounting to 2 mL. Figure S3. Reductive dechlorination of TCE in CO-fed enrichment cultures from transfer 2 to transfer 4. Approximately 33 μmol of TCE was dechlorinated to VC over 120 days in transfer 2 cultures (A). In transfer 3 cultures, 33 μmol of TCE was dechlorinated to VC with a small amount of ethene in 80 days (B). In transfer 4 cultures, 33 μmol of TCE was dechlorinated to ethene over 160 days (C). Red arrows indicate CO additions, with each dose amounting to 2 mL. Figure S4. Phylogenetic tree constructed based on the amino acid sequences of 43 RDases, with branches calculated from 500 bootstrap iterations. Functional assignments of these RDases were determined through biochemical characterization, expression analysis, or phylogenetic inference, representing a diverse array of OHRB. The tree also includes 20 [file 40168_2024_1869_MOESM1_ESM.docx]

**CO-Driven Electron and Carbon Flux Fuels Synergistic Microbial Reductive Dechlorination**

Jingjing Wang^1^, Xiuying Li^1^, Huijuan Jin^1^, Shujing Yang^1,3^, Lian Yu^4^, Hongyan Wang^1,2^, Siqi Huang^1,2^, Hengyi Liao^1,2^, Xuhao Wang^1,2^, Jun Yan^1^, Yi Yang^1, 5*^

^1^Key Laboratory of Pollution Ecology and Environmental Engineering, Institute of Applied Ecology, Chinese Academy of Sciences; Shenyang, Liaoning, 110016, China;

^2^University of Chinese Academy of Sciences, Beijing, 100049, China;

^3^Shenyang Pharmaceutical University, Shenyang, Liaoning, 117004, China;

^4^Department of Environmental Engineering, Beijing Institute of Petrochemical Technology, Beijing, 102617, China;

^5^Key Laboratory of Forest Ecology and Silviculture, Institute of Applied Ecology, Chinese Academy of Sciences, Shenyang, Liaoning, 110016, China.

***Corresponding author**

Yi Yang

Key Laboratory of Pollution Ecology and Environmental Engineering

Institute of Applied Ecology

Chinese Academy of Sciences

Shenyang, Liaoning, 110016, China

E-mail: yangyi@iae.ac.cn

Telephone: +86-13179657259

ORCID

Yi Yang: 0000-0002-3519-5472

Jingjing Wang: 0009-0008-0753-1732

Jun Yan: 0000-0001-6883-8529

Xiuying Li: 0000-0003-3555-7418

**MATERIALS AND METHODS**

**DNA extraction and 16S rRNA gene amplicon sequencing.**

Following the dechlorination of TCE to ethene, the cells were harvested from 5 mL culture suspension via vacuum filtration onto 25 mm diameter 0.22 μm pore-size membrane filters (Merck Millipore Ltd, Darmstadt, Germany). Genomic DNA was extracted from the filters using the Soil DNA Kit (Tiangen Biotech, Beijing, China) following the manufacturer’s instructions. DNA concentrations were determined using a Qubit 3.0 fluorometer (Invitrogen, Carlsbad, CA, USA). Amplicon sequencing was performed by Azenta Life Science (Suzhou, Jiangsu, China). A quantity of 20-30 ng of DNA was used to generate amplicons that cover V3 and V4 hypervariable regions of the 16S rRNA gene of prokaryotes, encompassing both bacteria and archaea. The primer pair Pro341F (5’-CCTACGGRRBGCASCAGKVRVGAAT-3’) and Pro806R (5’-GGACTACNVGGGTWTCTAATCC-3’) modified by Azenta were used to generate amplicons[1]. The 25 ul PCR mixture was prepared with 2.5 ul of TransStart buffer, 2 ul of dNTPs, 1 ul of each primer, 0.5 ul of TransStart Taq DNA polymerase and 20 ng template DNA. The PCR is performed by the following program: 3 min of denaturation at 94℃, 24 cycles of 5s at 95℃, 90s of annealing at 57℃, 10s of elongation at 72℃, and a final extension at 72℃ for 5min. Indexed adapters were added to the ends of the amplicons by limited cycle PCR. Finally, the library is purified with magnetic beads. The concentration is measured by a microplate reader (Tecan, Infinite 200 Pro) and the fragment size is detected by 1.5% agarose gel electrophoresis which is expected at ~600 bp. Next generation sequencing was conducted on an Illumina Miseq/Novaseq Platform (Illumina, San Diego, USA). Automated cluster generation and 250/300 paired-end sequencing with dual reads were performed according to the manufacturer’s instructions. Following quality control, sequences were grouped into operational taxonomic units (OTUs) using VSEARCH (v1.9.6) and QIIME (v1.9.1) at a 97% sequence identity. All OTUs were assigned to the lowest possible taxonomic rank using RDP classifier (Ribosomal Database Program) 2.2 and Silva_138 16S rRNA database [2, 3].

**PCR and sanger sequencing.**

Amplification of *Dehalococcoides* 16S rRNA gene was carried out using published primers Fp DHC 1 (5’GATGAACGCTAGCGGCG3’) and Rp DHC 1377 (5’GGTTGGCACATCGACTTCAA3’) and performed on a Veriti 96-well thermal cycler (Thermo Fisher Scientific, Waltham, MA, USA)[4]. The 25 μL PCR mixture contained 12.5 μL of Premix Taq master mix (Takara Bio Inc., Beijing, China), 2 μL of the DNA template, 1 μL each primer (10 μM), and 8.5 μL nuclease-free water. The PCR was performed by the following program: 2 min of denaturation at 94 ℃, 30 cycles of 30 s at 98 ℃, 30 s of annealing at 58 ℃, 2 min of elongation at 72 ℃, and a final extension at 72 ℃ for 10 min. The PCR products were checked using agarose gel (1.5%) electrophoresis, stained with GelRed. Sanger sequencing was performed by Azenta Inc (Nanjing, Jiangsu, China) using an Applied Biosystems 3730XL sequencer (Thermo Fisher Scientific) as described[5].

**Quantitative real-time PCR (qPCR) assay.**

Cells were collected from 1 mL culture suspensions via vacuum filtration onto 0.22 μm membrane filters (Jinteng, Tianjin, China) as described. Genomic DNA was extracted from the filters using the Soil DNA Kit (Tiangen Biotech, Beijing, China) following the manufacturer’s instructions. *Dhc* cell numbers were determined using a qPCR assay targeting the 16S rRNA gene. The qPCR assays were performed on a QuantStudio^TM^ 3 Real-Time PCR system (Applied Biosystems, Waltham, MA, USA) using primer set Dhc1200F (5’CTGGAGCTAATCCCCAAAGCT3’) and Dhc1271R (5’CAACTTCATGCAGGCGGG3’) and probe Dhc1240Probe (5’FAM-TCCTCAGTTCGGATTGCAGGCTGAA-TAMRA3’) [6] following established protocols. Briefly, each 25 μL assay contained 12.5 μL of 2× Premix Ex Taq master mix (Takara Bio Inc.), 200 nM each primer and probe, 0.25 μL of 50× ROX reference dye II (Takara Bio Inc.), 2 μL of the DNA template, and nuclease-free water. The cycle conditions were as follows: 95 °C for 30 s followed by 40 cycles of 5 s at 95 °C and 34 s at 60 °C. Standard curves were generated using three independent dilution series of plasmid DNA carrying a fragment *Dhc* 16S rRNA gene (GenBank accession number AY165308.1) [7]. Cell number calculations assumed one 16S rRNA gene copies per genome for *Dhc* .

**Phylogenetic Analysis.**

A collection of seventeen 16S rRNA gene sequences belonging to *Dehalococcoides*, representing three sub groups Pinellas (P), Cornell (C), and Victoria (V) [8], along with six 16S rRNA gene sequences from *Dehalogenimonas*, were retrieved from GenBank. The alignment of these sequences was conducted using MEGA version 10.2.4 software[9]. The ensuing phylogenetic tree was constructed using the Maximum Likelihood method and Tamura-Nei model [10], followed by 1000 bootstrap analysis. Additionally, protein sequences of functionally characterized reductive dehalogenases (RDases) from various OHRB were obtained from the Reductive Dehalogenase Database (https://rdasedb.biozone.utoronto.ca/), and putative RDases encoded in the genomes of *Dehalococcoides* sp. strain CO, assembled as part of this study, were included. Alignment of these sequences was performed using the Muscle plug-in in MEGA (version 10.2.4), and a phylogenetic tree was constructed using the maximum likelihood tree builder plug-in in MEGA with 500 bootstraps. The genome-aggregate average nucleotide identity (ANI) was calculated by EzBioCloud-ANI Calculator (<https://www.ezbiocloud.net/tools/ani>). The digital DNA-DNA hybridization (dDDH) was calculated by Genome-to-Genome Distance Calculator 2.1 (<https://www.dsmz.de/services/online-tools/genome-to-genome-distance-calculator-ggdc>).


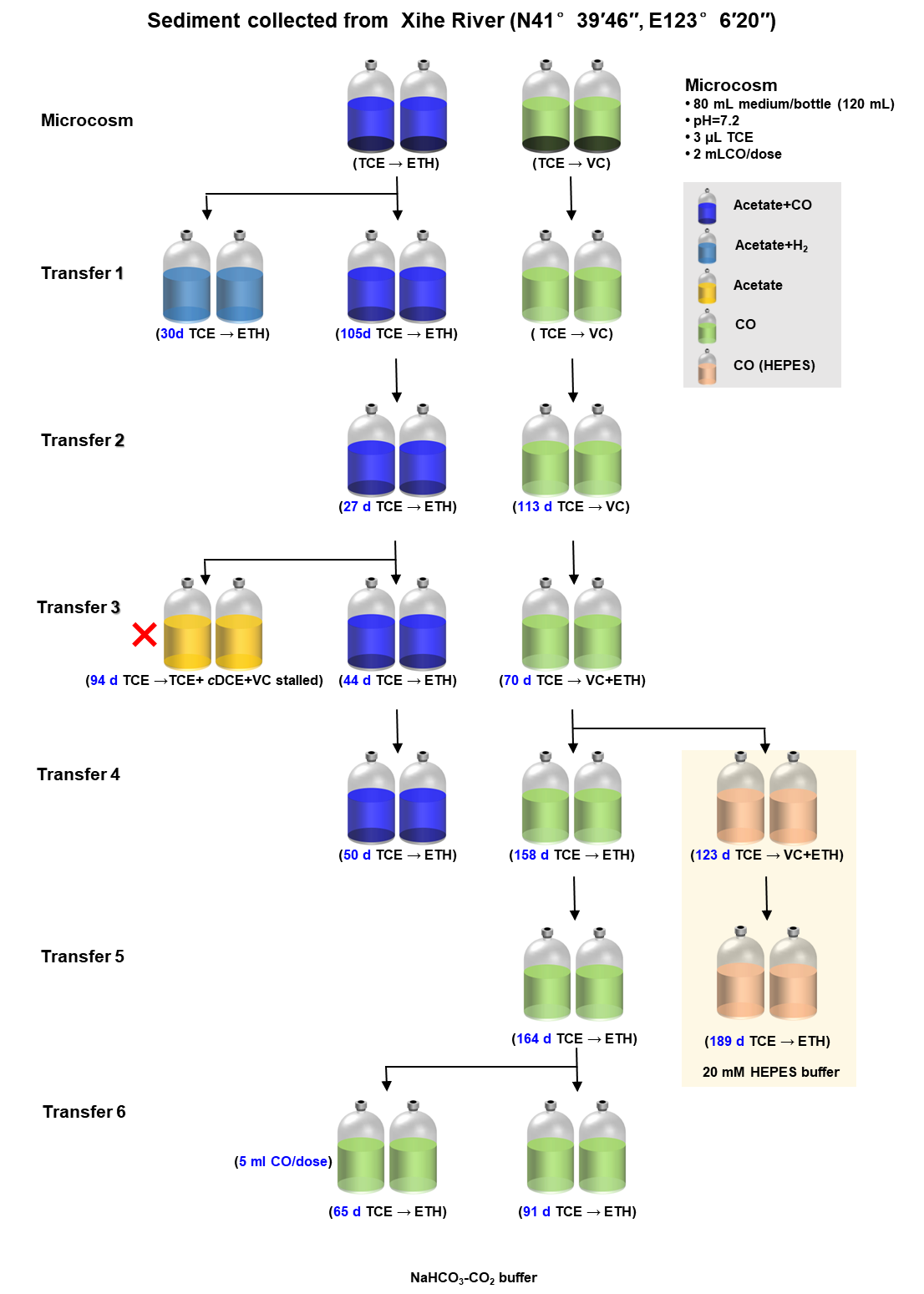


Figure S1 Microcosm setup and transferred enrichment cultures. Trichloroethylene (TCE) was dechlorinated in enrichment cultures fed with acetate and CO (represented by the royal blue bottles). Similarly, TCE dechlorination occurred in CO-fed enrichment cultures with bicarbonate-buffered medium (represented by the green bottles) and HEPES-buffered medium (represented by the orange bottles). Conversely, in acetate-fed enrichment cultures without CO or H_2_ supplementation (represented by the yellow bottles), the TCE dechlorination process did not occur, denoted by the symbol “X”, signifying the inability to dechlorinate.


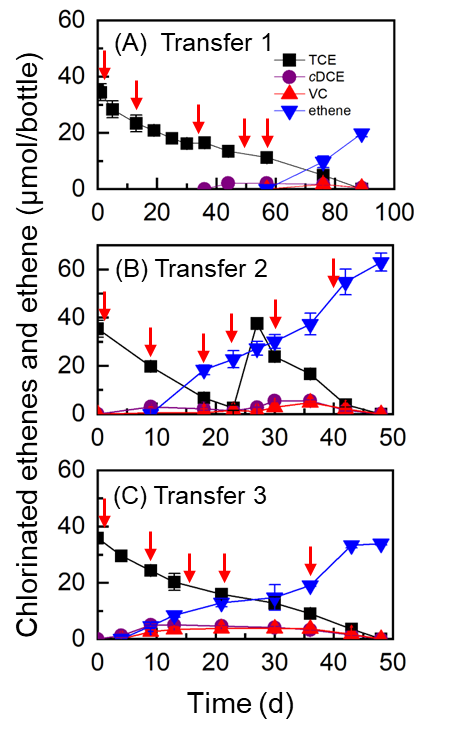


Figure S2 Reductive dechlorination of TCE in CO plus acetate enrichment cultures from transfer 1 to transfer 3. Approximately 33 μmol of TCE was dechlorinated to ethene over 100 days in transfer 1 cultures fed with acetate and CO (A). In transfer 2 cultures, 33 μmol of TCE was dechlorinated to ethene within 30 days, and an additional 33 μmol of TCE was dechlorinated within 25 days (B). In transfer 3 cultures, 33 μmol of TCE was dechlorinated to ethene over 50 days (C). Red arrows indicate CO additions, with each dose amounting to 2 mL.


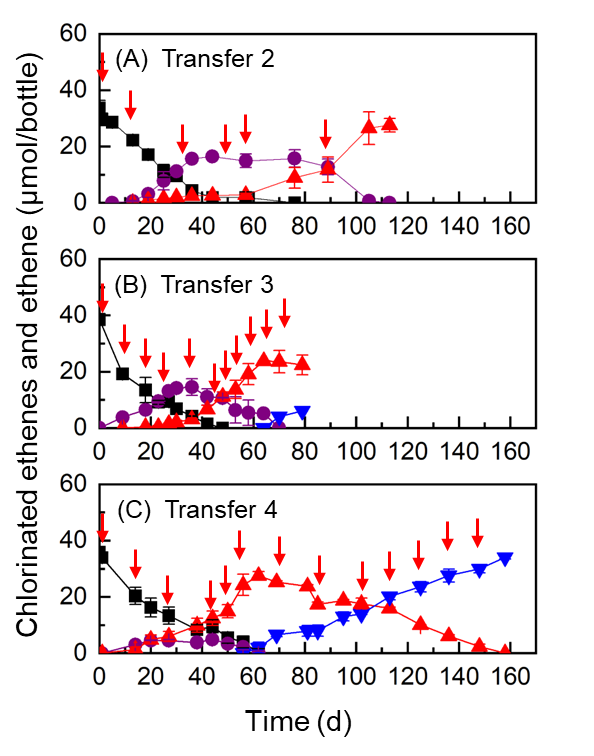


Figure S3 Reductive dechlorination of TCE in CO-fed enrichment cultures from transfer 2 to transfer 4. Approximately 33 μmol of TCE was dechlorinated to VC over 120 days in transfer 2 cultures (A). In transfer 3 cultures, 33 μmol of TCE was dechlorinated to VC with a small amount of ethene in 80 days (B). In transfer 4 cultures, 33 μmol of TCE was dechlorinated to ethene over 160 days (C). Red arrows indicate CO additions, with each dose amounting to 2 mL.


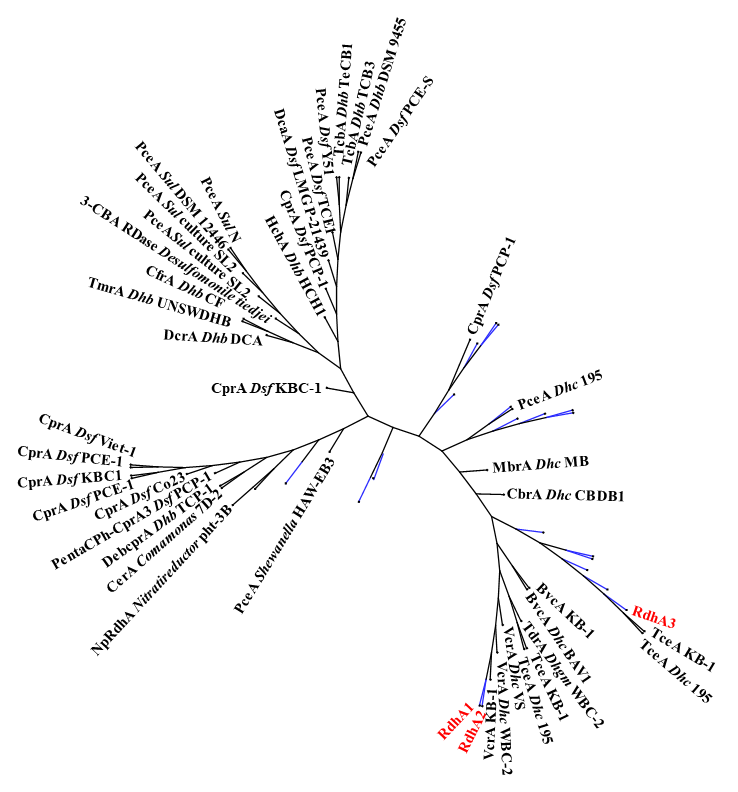


Figure S4 Phylogenetic tree constructed based on the amino acid sequences of 43 RDases, with branches calculated from 500 bootstrap iterations. Functional assignments of these RDases were determined through biochemical characterization, expression analysis, or phylogenetic inference, representing a diverse array of OHRB. The tree also includes 20 RDases annotated from the draft genome of strain CO, highlighted in blue branches.


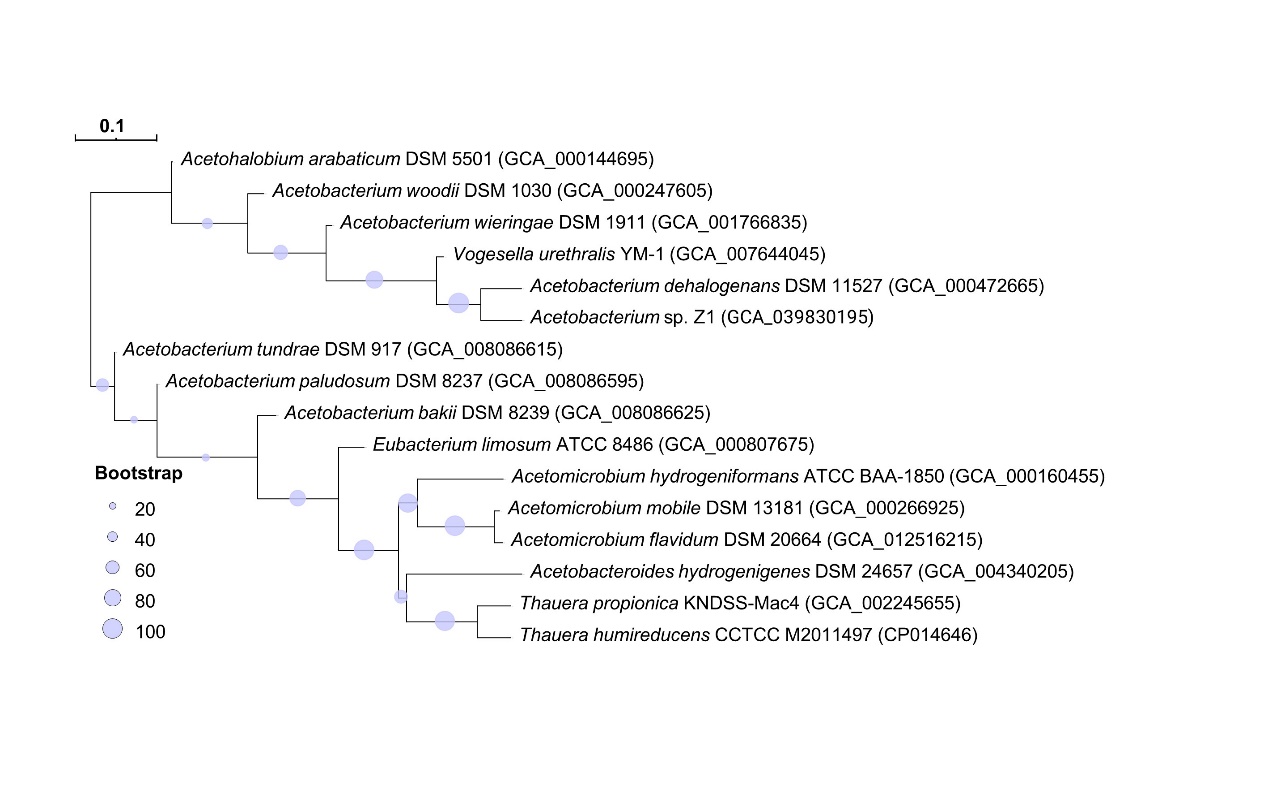


Figure S5 Phylogeny of *Acetobacterium* based on genome sequences. The tree was constructed using the maximum-likelihood method, with GenBank accession numbers provided in parentheses. Bootstrap values, derived from 1,000 resamplings, are indicated at branching points.


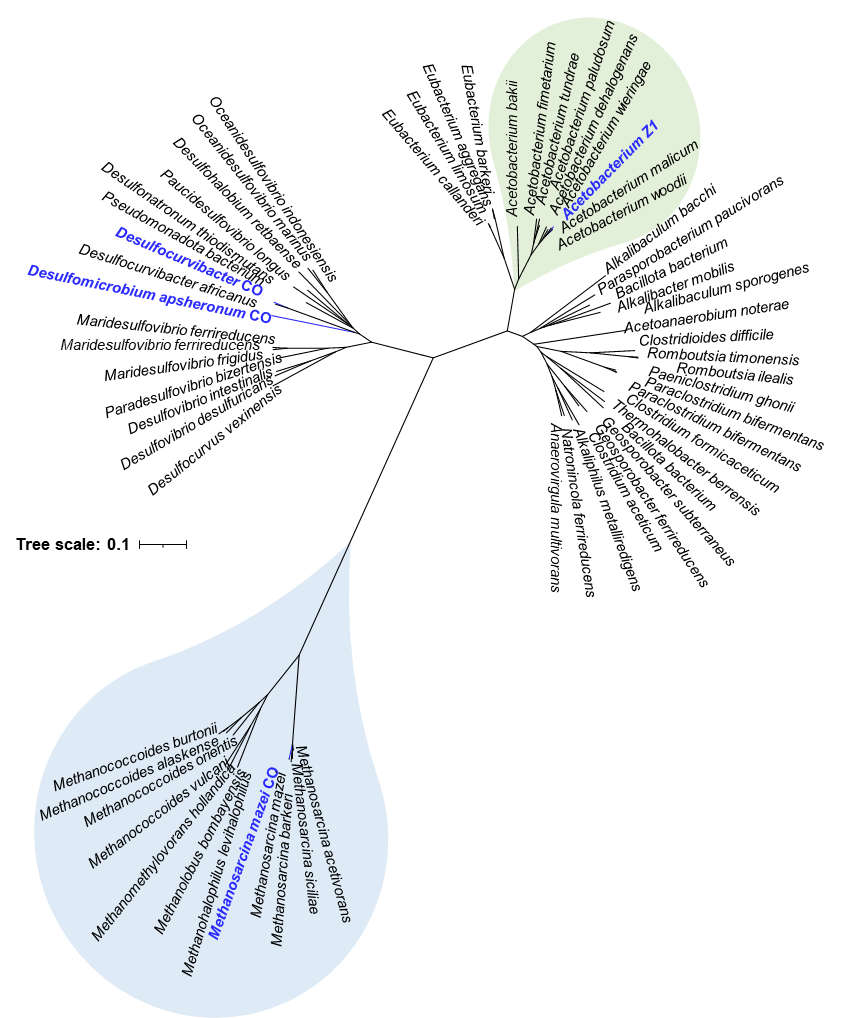


Figure S6 Phylogenetic tree based on protein sequences of 63 carbon monoxide dehydrogenases (CODHs) from diverse bacteria and methanogens (archaea). CODHs from methanogens are indicated with a blue background, those from Acetobacterium with a green background, and CODHs identified in CO enrichment cultures are highlighted in blue.


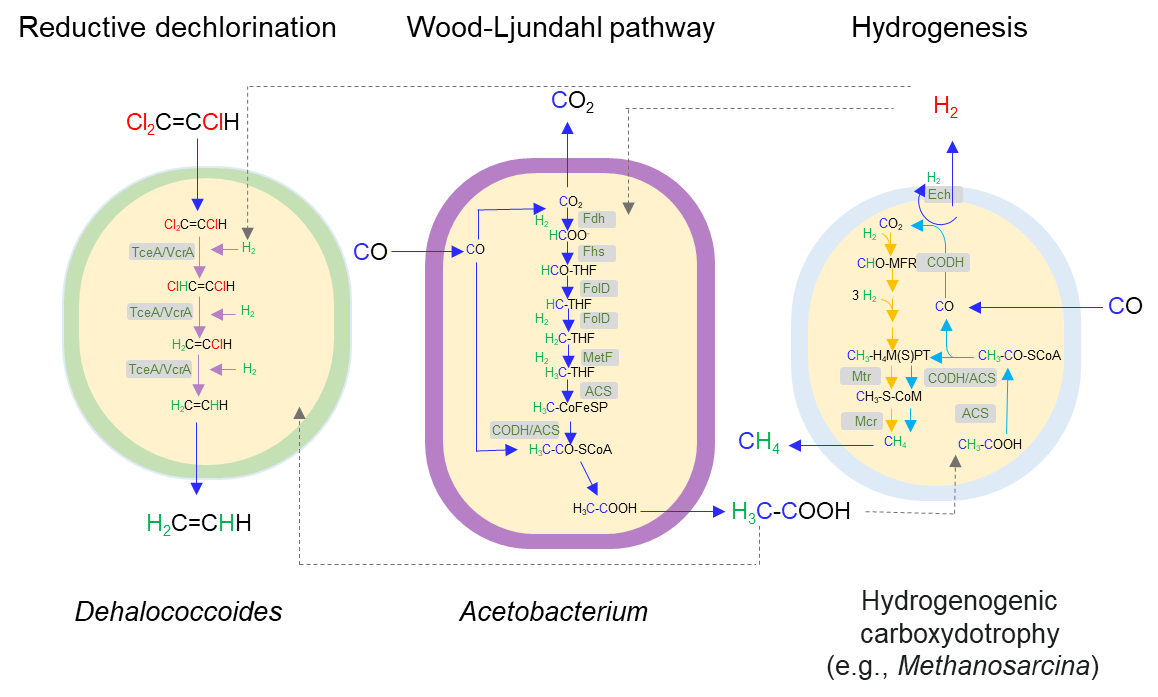


Figure S7 Proposed model for interspecies interactions supporting TCE-to-ethene dechlorination by *Dehalococcoides* with CO as electron donor and carbon source. Hydrogenogenic carboxydotrophs (e.g., *Methanosarcina*) oxidize CO, generating H₂. This H₂ is then transferred to *Acetobacterium*, which in turn produces acetate. Both acetate and H₂ are subsequently utilized by *Dehalococcoides* for reductive dechlorination of TCE to ethene. Additionally, *Methanosarcina* can use acetate and H₂ to produce methane.

Table S1 The average nucleotide identity (%) and digital DNA-DNA hybridization (%) of strain CO with other strains in the genus of *Dehalococcoides*

| **Strains of *Dehalococcoides*** | **G+C（%）** | **The average nucleotide identity (ANI) (%) and** **digital DNA-DNA hybridization (dDDH) (%)** | | | | | | | | |
| --- | --- | --- | --- | --- | --- | --- | --- | --- | --- | --- |
|  |  | 1 | 2 | 3 | 4 | 5 | 6 | 7 | 8 | 9 |
| 1.*Dhc* CO | 47.2 | _ | 30.6 | 99.6 | 96.4 | 98.1 | 31.3 | 95.4 | 32.6 | 93.9 |
| 2. *Dhc* 195 | 48.8 | 86.3 | _ | 30.3 | 30.3 | 30.5 | 38.2 | 29.5 | 38.2 | 30.6 |
| 3. *Dhc* FL2 | 46.9 | 99.7 | 86.1 | _ | 97.7 | 92.8 | 31.1 | 95.2 | 31.3 | 91.0 |
| 4. *Dhc* CBDB1 | 47.0 | 99.3 | 86.1 | 99.6 | _ | 94.5 | 30.8 | 96.4 | 31.1 | 94.0 |
| 5. *Dhc* 11a5 | 46.9 | 99.4 | 86.1 | 99.1 | 99.5 | _ | 31.3 | 95.2 | 31.1 | 31.9 |
| 6. *Dhc* VS | 47.3 | 86.8 | 89.9 | 86.7 | 86.6 | 86.8 | _ | 31.2 | 77.7 | 89.9 |
| 7. *Dhc* KS | 47.6 | 99.4 | 85.8 | 99.3 | 99.6 | 99.3 | 86.8 | _ | 31.6 | 93.5 |
| 8. *Dhc* CG3 | 46.9 | 87.7 | 89.5 | 87.0 | 86.4 | 86.3 | 97.6 | 87.2 | _ | 32.4 |
| 9. *Dhc* 11a | 47.6 | 98.9 | 86.1 | 98.6 | 99.3 | 98.6 | 87.0 | 99.2 | 87.7 | _ |

Table S2 RDases with assigned functions and their host OHRB used in Figure S4.

| RDase | Host OHRB | GenBank accession number |
| --- | --- | --- |
| VcrA | *Dhc* strain VS | WP_012882535 |
| VcrA | *Dhc* strain WBC-2 | AOV99943 |
| VcrA | KB-1 consortium | AQY73737 |
| TmrA | *Dhb* strain UNSWDHB | WP_034377773 |
| TdrA | *Dhgm* strain WBC-2 | AKG53095 |
| TceA | *Dhc* strain 195 | AAW39214 |
| TceA | KB-1 consortium | WP_078987038 |
| TceA | KB-1 consortium | AIZ97109 |
| TceA | *Dhc* strain 195 | AAW39060 |
| TcbA | *Dhb* strain TeCB1 | WP_068882928 |
| TcbA | *Dhb* strain TCB3 | 2821531494 |
| PentaCPh-CprA3 | *Dsf* strain PCP-1 | AAK06764 |
| PceA | *Dhc strain* 195 | AAW40342 |
| PceA | *Sul* strain DSM 12446 | AHJ12791 |
| PceA | *Dsf* strainTCE1 | CAD28792 |
| PceA | *Dsf* strainY51 | WP_011460641 |
| PceA | *Sul* culture SL2 | AGW23615 |
| PceA | *Sul* culture SL2 | AGW23613 |
| PceA | *Sul* strain N | AAC60788 |
| PceA | *Dhb* strainDSM 9455 | AHF10727 |
| NpRdhA | *Nitratireductor* strain pht-3B | EKF18105 |
| MbrA | *Dhc* strain MB | ADF96893 |
| HchA | *Dhb* strain HCH1 | 2823894057 |
| DebcprA | *Dhb* strain TCP-1 | AGC09147 |
| DcrA | *Dhb* strain DCA | AFV02209 |
| DcaA | *Dsf* strain LMG P-21439 | CAJ75430 |
| CprA | *Dsf* strain PCP-1 | AAK95329 |
| CprA | *Dsf* strain Co23 | AAL84925 |
| CprA | *Dsf* strain KBC1 | AB194705 |
| CprA | *Dsf* strain PCE-1 | AAG49543 |
| CprA | *Dsf* strain PCE-1 | AAG46187 |
| CprA | *Dsf* strain Viet-1 | AAG49544 |
| CprA | *Dsf* strain KBC-1 | BAE45338 |
| CprA | *Dsf* strain PCP-1 | AAQ54585 |
| CfrA | *Dhb* strain CF | AFV05253 |
| CerA | *Comamonas* strain 7D-2 | AFV28965 |
| CbrA | *Dhc* strain CBDB1 | CAI82345 |
| BvcA | *Dhc* strain BAV1 | AAT64888 |
| BvcA | KB-1 consortium | ABA64533 |
| 3-CBA RDase | *Desulfomonile tiedjei* | AFM24124 |
| **RdhA1** | ***Dhc* strain CO** | **PP060998 (This study)** |
| **RdhA2** | ***Dhc* strain CO** | **PP060999 (This study)** |
| **RdhA3** | ***Dhc* strain CO** | **PP061000 (This study)** |

OHRB abbreviations: *Dhc*, *Dehalococcoides mccartyi*; *Dhgm*, *Dehalogenimonas*; *Dhb*, *Dehalobacter*; *Dsf*, *Desulfitobacterium*; *Sul*, *Sulfurospirillum*.

Table S3 The average nucleotide identity (%) and digital DNA-DNA hybridization (%) of strain Z1 with other strains in the genus of *Acetobacterium*

| **Strains of *Acetobacterium*** | **G+C（%）** | **The average nucleotide identity (ANI) (%) and** **digital DNA-DNA hybridization (dDDH) (%)** | | | | | | | | | | | |
| --- | --- | --- | --- | --- | --- | --- | --- | --- | --- | --- | --- | --- | --- |
|  |  | 1 | 2 | 3 | 4 | 5 | 6 | 7 | 8 | 9 | 10 | 11 | 12 |
| 1. *A*. sp. Z1 | 44.5 | _ | 21.4 | 37.7 | 19.6 | 19.4 | 27.4 | 28.0 | 19.5 | 21.2 | 36.9 | 19.6 | 19.3 |
| 2. *A*. *woodii* DSM 1030 | 39.3 | 77.4 | _ | 21.9 | 20.5 | 21.6 | 21.3 | 21.5 | 21.6 | 20.7 | 21.9 | 20.5 | 20.5 |
| 3. *A*. *dehalogenans* DSM 11527 | 43.8 | 89.2 | 77.3 | _ | 19.8 | 16.1 | 27.2 | 27.5 | 19.9 | 21.3 | 71.9 | 16.0 | 20.3 |
| 4. *A*. *paludosum* DSM 8237 | 40.0 | 75.1 | 74.5 | 75.1 | _ | 61.5 | 19.6 | 19.9 | 61.5 | 24.2 | 19.9 | 100.0 | 21.3 |
| 5. *A*. *tundrae* DSM 917 | 39.6 | 75.2 | 75.0 | 75.2 | 95.2 | _ | 19.4 | 19.7 | 100 | 23.7 | 20.2 | 61.5 | 20.9 |
| 6. A. *wieringae* JM | 44.3 | 83.5 | 75.6 | 83.4 | 74.3 | 74.3 | _ | 76.1 | 19.5 | 20.5 | 27.5 | 19.6 | 19.4 |
| 7. *A*. *wieringae* Y | 44.2 | 84.0 | 76.8 | 83.5 | 74.2 | 74.5 | 97.3 | _ | 19.7 | 20.5 | 27.5 | 19.9 | 19.8 |
| 8.*A*. *tundrae* DER-2019 | 39.7 | 75.0 | 75.6 | 75.2 | 95.2 | 99.96 | 74.4 | 74.4 | _ | 23.7 | 20.0 | 61.5 | 20.9 |
| 9. *A*. *fimetarium* DER-2019 | 44.7 | 75.7 | 74.0 | 75.8 | 80.0 | 79.7 | 75.1 | 74.6 | 79.7 | _ | 21.4 | 24.2 | 21.0 |
| 10. *A*. *malicum* DER-2019 | 43.7 | 88.7 | 77.7 | 95.6 | 75.0 | 75.2 | 83.6 | 83.3 | 75.3 | 75.7 | _ | 19.9 | 21.2 |
| 11. *A*. *paludosum* DER-2019 | 40.1 | 75.1 | 75.0 | 75.0 | 99.96 | 95.1 | 74.3 | 74.2 | 95.1 | 80.0 | 75.0 | _ | 21.3 |
| 12. *A. bakii* DSM 8239 | 41.2 | 74.6 | 74.3 | 74.9 | 75.9 | 75.4 | 74.1 | 74.2 | 75.4 | 74.9 | 75.5 | 75.8 | _ |

Table S4 Metagenome-assembled genomes recovered from CO-fed TCE-dechlorinating enrichment cultures.

| **Bins** | **Completeness** | **Contamination** | **Domain** | **Phylum** | **Class** | **Order** | **Family** | **Genus** |
| --- | --- | --- | --- | --- | --- | --- | --- | --- |
| 1 | 99.13 | 0.82 | Bacteria | *Bacteroidota* | *Bacteroidia* | *Bacteroidales* | *Dysgonomonadaceae* | *Petrimonas* |
| 2 | 97.91 | 0.85 | Archaea | *Halobacteriota* | *Methanomicrobia* | *Methanomicrobiales* | *Methanofollaceae* | *Methanofollis* |
| 3 | 97.92 | 1.75 | Bacteria | *Actinobacteriota* | *Coriobacteriia* | OPB41 | UBA2279 | UBA7930 |
| 4 | 99.01 | 0 | Bacteria | *Chloroflexota* | *Dehalococcoidia* | *Dehalococcoidales* | *Dehalococcoidaceae* | *Dehalococcoides* |
| 5 | 99.05 | 2.62 | Bacteria | *Bacteroidota* | *Bacteroidia* | *Bacteroidales* | VadinHA17 | SR-FBR-E99 |
| 6 | 99.96 | 0 | Bacteria | *Desulfobacterota* | *Desulfovibrionia* | *Desulfovibrionales* | *Desulfomicrobiaceae* | *Desulfomicrobium* |
| 7 | 99.3 | 2.12 | Bacteria | *Firmicutes* | *Clostridia* | *Tissierellales* | *Tissierellaceae* | *Gudongella* |
| 8 | 98.84 | 1.1 | Bacteria | *Cloacimonadota* | *Cloacimonadia* | *Cloacimonadales* | *Cloacimonadaceae* |  |
| 9 | 99.3 | 0.04 | Bacteria | *Firmicutes* | *Clostridia* | *Eubacteriales* | *Eubacteriaceae* | *Acetobacterium* |
| 10 | 100 | 0 | Bacteria | *Synergistota* | *Synergistia* | *Synergistales* | *Aminobacteriaceae* | *Aminivibrio* |
| 11 | 89.65 | 2.63 | Bacteria | *Firmicutes* | *Peptococcia* | DRI-13 | UBA5745 |  |
| 12 | 99.7 | 0 | Bacteria | *Desulfobacterota* | *Desulfovibrionia* | *Desulfovibrionales* | *Desulfovibrionaceae* | *Desulfocurvibacter* |
| 13 | 84.1 | 2.69 | Bacteria | *Firmicutes* | *Clostridia* | *Clostridiales* | *Clostridiaceae* | *Youngiibacter* |
| 14 | 96.45 | 0 | Bacteria | *Desulfobacterota* | *Desulfovibrionia* | *Desulfovibrionales* | *Desulfovibrionaceae* | *Aminidesulfovibrio* |
| 15 | 84.28 | 1.19 | Bacteria | *Desulfobacterota* | *Desulfovibrionia* | *Desulfovibrionales* | *Desulfovibrionaceae* | *Solidesulfovibrio* |
| 16 | 99.19 | 0.81 | Bacteria | *Firmicutes* | *Clostridia* | *Christensenellales* | *UBA6094* | *UBA6094* |
| 17 | 98.1 | 0.65 | Archaea | *Halobacteriota* | *Methanomicrobia* | *Methanomicrobiales* | *Methanoculleaceae* | *Methanoculleus* |
| 18 | 98.6 | 0.93 | Bacteria | *Firmicutes* | *Clostridia* | *Tissierellales* | *Dethiosulfatibacteraceae* | *Nov-38* |
| 19 | 96.73 | 0.65 | Archaea | *Halobacteriota* | *Methanosarcinia* | *Methanosarcinales* | *Methanosarcinaceae* | *Methanosarcina* |
| 20 | 97.36 | 0.14 | Bacteria | *Firmicutes* | *Thermincolia* | *Thermincolales* | UBA2595 |  |
| 21 | 90 | 1.61 | Bacteria | *Bacteroidota* | *Bacteroidia* | *Bacteroidales* | *Lentimicrobiaceae* | *Lentimicrobium* |
| 22 | 98.85 | 0 | Bacteria | *Spirochaetota* | *Spirochaetia* | *Treponematales* | UBA8932 | UBA2256 |
| 23 | 95.24 | 1.18 | Bacteria | *Desulfobacterota* | *Desulfovibrionia* | *Desulfovibrionales* | *Desulfovibrionaceae* | UBA6814 |
| 24 | 99.32 | 5.06 | Bacteria | *Firmicutes* | *Negativicutes* | UBA1444 | UBA1444 | UBA1444 |
| 25 | 97.08 | 1.97 | Bacteria | *Firmicutes* | *Bacilli* | *Bacillales* | *Bacillaceae* | *Calidifontibacillus* |

**REFERENCE**

1. Fu S-F, Wang F, Shi X-S, Guo R-B. Impacts of microaeration on the anaerobic digestion of corn straw and the microbial community structure*.* Chem Eng J. 2016; 287: 523-28.

2. Edgar RC. Search and clustering orders of magnitude faster than blast*.* Bioinformatics. 2010; 26(19): 2460-1.

3. Wang Q, Garrity George M, Tiedje James M, Cole James R. Naïve bayesian classifier for rapid assignment of rrna sequences into the new bacterial taxonomy*.* Appl Environ Microbiol. 2007; 73(16): 5261-67.

4. Hendrickson ER, Payne JA, Young RM, Starr MG, Perry MP, Fahnestock S*,* et al*.* Molecular analysis of *Dehalococcoides* 16s ribosomal DNA from chloroethene-contaminated sites throughout north america and europe*.* Appl Environ Microbiol. 2002; 68(2): 485-95.

5. Walker SE, Lorsch J. Chapter fourteen - sanger dideoxy sequencing of DNA, in *Methods in enzymology*, Lorsch J, Editor. 2013, Academic Press. 171-84.

6. He J, Ritalahti KM, Aiello MR, Löffler FE. Complete detoxification of vinyl chloride by an anaerobic enrichment culture and identification of the reductively dechlorinating population as a *Dehalococcoides* species*.* Appl Environ Microbiol. 2003; 69(2): 996-1003.

7. Jiang L, Yang Y, Jin H, Wang H, Swift CM, Xie Y*,* et al*.* *Geobacter* sp. strain IAE dihaloeliminates 1,1,2-trichloroethane and 1,2-dichloroethane*.* Environ Sci Technol. 2022; 56(6): 3430-40.

8. Löffler FE, Yan J, Ritalahti KM, Adrian L, Edwards EA, Konstantinidis KT, et al*.* *Dehalococcoides mccartyi* gen. nov., sp. nov., obligately organohalide-respiring anaerobic bacteria relevant to halogen cycling and bioremediation, belong to a novel bacterial class, *Dehalococcoidia* classis nov., order *Dehalococcoidales* ord. nov. and family *Dehalococcoidaceae* fam. nov., within the phylum *Chloroflexi.* Int J Syst Evol Micr. 2015; 65(Pt_6): 2015-15.

9. Kumar S, Stecher G, Li M, Knyaz C, Tamura K. Mega X: Molecular evolutionary genetics analysis across computing platforms*.* Mol Biol Evol. 2018; 35(6): 1547-49.

10. Tamura K, Nei M. Estimation of the number of nucleotide substitutions in the control region of mitochondrial DNA in humans and chimpanzees*.* Mol Biol Evol. 1993; 10(3): 512-26.
